# Supplementary material for: Person-centred eHealth intervention for patients on sick leave due to common mental disorders: study protocol of a randomised controlled trial and process evaluation (PROMISE)
Source: BMJ Open. 2020 Sep 1;10(9):e037515. doi: 10.1136/bmjopen-2020-037515 (PMC7467509; doi:10.1136/bmjopen-2020-037515)
Supplement: Author's manuscript [file bmjopen-2020-037515.draft_revisions.pdf]

BMJ Open

BMJ Open is committed to open peer review. As part of this commitment we make the peer review history of every article we publish publicly available.

When an article is published we post the peer reviewers' comments and the authors' responses online. We also post the versions of the paper that were used during peer review. These are the versions that the peer review comments apply to.

The versions of the paper that follow are the versions that were submitted during the peer review process. They are not the versions of record or the final published versions. They should not be cited or distributed as the published version of this manuscript.

BMJ Open is an open access journal and the full, final, typeset and author-corrected version of record of the manuscript is available on our site with no access controls, subscription charges or pay-per-view fees (http://bmjopen.bmj.com).

If you have any questions on BMJ Open's open peer review process please email info.bmjopen@bmj.com

BMJ Open

A person-centred eHealth intervention for patients on sick-leave due to common mental disorders: Protocol of a randomised controlled trial and process evaluation (PROMISE)

Journal:	*BMJ Open*
Manuscript ID	bmjopen-2020-037515
Article Type:	Protocol
Date Submitted by the Author:	05-Feb-2020

Complete List of Authors:	Cederberg, Matilda; University of Gothenburg Institute of Health and Care Sciences, Centre for Person-Centred Care, Sahlgrenska Academy<br>Ali, Lilas; University of Gothenburg Institute of Health and Care Sciences, Centre for Person-Centred Care, Sahlgrenska Academy; Sahlgrenska University Hospital, Department of Psychiatry<br>Ekman, Inger; University of Gothenburg Institute of Health and Care Sciences, Centre for Person-Centred Care, Sahlgrenska Academy; Sahlgrenska University Hospital, Department of Internal medicine and geriatrics<br>Glise, Kristina; Institute of Stress Medicine<br>Jonsdottir, Ingibjörg; Institute of Stress Medicine; University of Gothenburg Sahlgrenska Academy, School of Public Health and Community medicine<br>Gyllensten, Hanna; University of Gothenburg Institute of Health and Care Sciences, Centre for Person-Centred Care, Sahlgrenska Academy<br>Swedberg, Karl; University of Gothenburg Institute of Health and Care Sciences, Centre for Person-Centred Care, Sahlgrenska Academy; University of Gothenburg Sahlgrenska Academy, Department of Molecular and Clinical Medicine<br>Fors, Andreas; University of Gothenburg Institute of Health and Care Sciences, Centre for Person-Centred Care, Sahlgrenska Academy; Närhälsan Research and Development Primary Health Care

Keywords:	MENTAL HEALTH, PRIMARY CARE, PUBLIC HEALTH, Information technology < BIOTECHNOLOGY & BIOINFORMATICS, Quality in health care < HEALTH SERVICES ADMINISTRATION & MANAGEMENT

SCHOLARONE™
Manuscripts

I, the Submitting Author has the right to grant and does grant on behalf of all authors of the Work (as defined in the below author licence), an exclusive licence and/or a non-exclusive licence for contributions from authors who are: i) UK Crown employees; ii) where BMJ has agreed a CC-BY licence shall apply, and/or iii) in accordance with the terms applicable for US Federal Government officers or employees acting as part of their official duties; on a worldwide, perpetual, irrevocable, royalty-free basis to BMJ Publishing Group Ltd ("BMJ") its licensees and where the relevant Journal is co-owned by BMJ to the co-owners of the Journal, to publish the Work in this journal and any other BMJ products and to exploit all rights, as set out in our licence.

The Submitting Author accepts and understands that any supply made under these terms is made by BMJ to the Submitting Author unless you are acting as an employee on behalf of your employer or a postgraduate student of an affiliated institution which is paying any applicable article publishing charge ("APC") for Open Access articles. Where the Submitting Author wishes to make the Work available on an Open Access basis (and intends to pay the relevant APC), the terms of reuse of such Open Access shall be governed by a Creative Commons licence – details of these licences and which Creative Commons licence will apply to this Work are set out in our licence referred to above.

Other than as permitted in any relevant BMJ Author's Self Archiving Policies, I confirm this Work has not been accepted for publication elsewhere, is not being considered for publication elsewhere and does not duplicate material already published. I confirm all authors consent to publication of this Work and authorise the granting of this licence.

For peer review only

A person-centred eHealth intervention for patients on sick-leave due to common mental disorders: Protocol of a randomised controlled trial and process evaluation (PROMISE).

Matilda Cederberg[1,2], Lilas Ali[1,2,3], Inger Ekman[1,2,4], Kristina Glise[5], Ingibjörg H Jonsdottir[5,6] Hanna Gyllensten[1,2], Karl Swedberg[1,2,4,7], Andreas Fors[1,2,8]

1 Institute of Health and Care Sciences, Sahlgrenska Academy, University of Gothenburg, Gothenburg, Sweden.
2 Centre for Person-Centred Care (GPCC), University of Gothenburg, Gothenburg, Sweden.
3 Sahlgrenska University Hospital, Psychiatric department, Gothenburg, Sweden.
4 Sahlgrenska university hospital/Östra/Department of Internal medicine and geriatrics, Gothenburg, Sweden.
5 The Institute of Stress Medicine at the Västra Götaland region, Gothenburg, Sweden
6 School of Public Health and Community Medicine, Sahlgrenska Academy, University of Gothenburg, Gothenburg, Sweden
7 Department of Molecular and Clinical Medicine, Sahlgrenska Academy, University of Gothenburg, Gothenburg, Sweden.
8 Närhälsan Research and Development Primary Health Care, Region Västra Götaland, Sweden.

Corresponding author: Matilda Cederberg, Institute of Health Care Sciences, Centre for Person-Centred Care, Sahlgrenska Academy, Box 457, 405 30 Gothenburg, Sweden.
matilda.cederberg@gu.se

Abstract

Introduction The number of people dealing with common mental disorders (CMDs) is a major concern in many countries, including Sweden. Sickness absence resulting from CMDs is often long-lasting and advancing return to work is a complex process impacted by several factors, among which self-efficacy appears to be an important personal resource. Developing health care strategies that build on person-centred care (PCC) is needed to advance recovery through collaboration and enhanced self-efficacy.

Methods and analysis This study is an open randomised controlled trial comparing a control group receiving standard care with an intervention group receiving standard care plus person-

For peer review only

centred care by eHealth and telephone. The primary outcome measure is a composite score of changes in sick leave and self-efficacy. Participants will include 220 primary care patients on sick leave due to CMDs and data will mainly be collected through questionnaires at baseline and 3, 6, 12 and 24 months from the inclusion date. Inclusion is ongoing and expected to be completed during the fall of 2020. A process and health economic evaluation will also be conducted.

Ethics and dissemination This study was approved by the Regional Ethical Review Board in Gothenburg, Sweden. Results will be published in peer-reviewed scientific journals and presented at national and international scientific conferences. This project is part of a broader research programme conducted at the Gothenburg Centre for Person-Centred Care, where extensive work is undertaken to disseminate knowledge on and implementation of PCC.

For peer review only

Article Summary

Strengths and limitations of this study

- A multiple-component, flexible intervention based on person-centred principles to facilitate communication and collaboration with the patient, their workplace and significant others.
- Besides a randomised controlled trial, we will conduct a health economic and process evaluation to obtain results on cost-effectiveness and the quantity and quality of the intervention implementation.
- The study includes patients with various diagnoses under the common mental disorders umbrella. The intervention has a generic design which makes it applicable also to other conditions, and to various categories of healthcare professionals.

For peer review only - http://bmjopen.bmj.com/site/about/guidelines.xhtml

- Because the study is mainly conducted in a research centre, the study results should be treated with this in mind and future adaptions may be needed before implementing the intervention in other contexts and settings.

Introduction

As a major determinant of short- and long-term sick leave in many countries, mental illness bears a substantial portion of health care expenses and challenges (1-3). In Sweden, the total cost of mental illness has been estimated at 7.7 billion euros annually, of which about 50% is attributable to direct medical costs and 50% to work absence and out-of-work benefits (1). The number of sick leaves with origins from mental illness has increased during the past decade, and it is now the most common cause of sick leave in Sweden (4, 5). The conditions most frequently causing sick leave are depression, anxiety and stress reactions and disorders, which are generally referred to as common mental disorders (CMDs) (2, 6, 7). Sick leave attributable to CMDs has a long mean duration, with high risk of recurrent sickness episodes (7, 8). In Sweden, the majority of consultations concerning CMDs take place in primary care, and most patients are treated within primary care (1, 9). First-line treatment often consists of cognitive behavioural therapy, medication, or both (10), but the relationship between sickness duration and return to work (RTW) is complex (11-14). The existing evidence on interventions affecting RTW is limited; however, interventions that include a workplace component are more successful in improving RTW (15-19). In addition, interventions with multiple components appear more successful than single-component interventions (19). A recent systematic review by Etuknwa et al (20) evaluated which personal and social factors impacted RTW and found that self-efficacy, a positive attitude and support from leaders and co-workers were most relevant. The possible influence of self-

For peer review only - http://bmjopen.bmj.com/site/about/guidelines.xhtml

efficacy on RTW has also been supported elsewhere (21, 22). Self-efficacy as a concept is rooted in social cognitive theory, which draws conclusions on human agency and its internal and external determinants (23). Perceived self-efficacy is a personal judgment of an individual's capacity to handle general or specific situations in life, and as such, it is a core function of human agency. It is assumed to be shaped by different sources of information and contextual factors; however, the best way to influence a person's self-efficacy is through successful experiences of mastery (24). Moreover, a strong sense of self-efficacy can reduce vulnerability to stressors and increase resilience to cope with adverse events (23, 25).

Enabling self-efficacy is a central goal in the Gothenburg Centre for Person-Centred Care (GPCC) approach to person-centred care (PCC) (26-29). PCC can be conceptualised as initiatives advocating that healthcare systems need to treat and understand patients as persons (30). The concept of person is of vital importance in PCC as it transcends the more or less temporary and contextual role of being a patient. Persons have many features, characteristics and abilities in common, such as the ability to communicate and self-reflect, and at the same time, each person is someone in his or her own right with particular personal and circumstantial bodies, characteristics, abilities and histories (31, 32). For health care professionals (HCPs) to know their patients as persons, they need to be receptive to the unique premises of their patients' life. This approachability includes being sensitive to what is important to the patients, and neither diminish them on account of their vulnerability nor abandon them on account of their capabilities (30). To support HCPs and organisations to enhance person-centredness in clinical practice GPCC proposed a framework to operationalise the ethics of PCC (29). In the framework a key factor in conducting PCC is the quality of communication and relationship, referred to as a partnership that includes evidence-based diagnostic procedures. Such a partnership is characterized by mutuality,

For peer review only - http://bmjopen.bmj.com/site/about/guidelines.xhtml

respect, sharing and understanding, and is, at the same time, achieved through these means. Hence, a partnership is both the goal and a means to achieve PCC.

Previous evaluations of the GPCC approach in various conditions and care contexts have shown positive effects on several outcomes, including health-related quality of life (HRQoL) (33), patients' care experiences (34), uncertainty in illness (35), care costs (36, 37) and self-efficacy (26, 27). However, this approach has not yet been evaluated for CMDs. Combining PCC with an eHealth tool was shown to enhance further the ability to improve self-efficacy (38), and positive effects on self-efficacy have been demonstrated using telephone support (28). In addition, it appears that physical encounters are not essential to develop a patient-professional partnership (28, 39). Telehealth and eHealth solutions challenge the traditional structures in health care and hold possibilities of increasing transparency and accessibility. eHealth services may increase the possibility of patient involvement (e.g., empowerment and shared-decision making (40)), and psychological or behavioural eHealth interventions may, for example, contribute to a greater reach of patients who otherwise might not be subject to such assistance (41). This paper presents the design of a study evaluating a person-centred eHealth intervention for patients on sick leave due to CMDs. The primary aim is to evaluate the effects of a person-centred eHealth intervention (digital platform and telephone support) for patients on sick leave due to CMDs. Secondary aims are to evaluate the cost-effectiveness and the process of implementation to assess the fidelity of the eHealth intervention.

Methods and analysis

For peer review only - http://bmjopen.bmj.com/site/about/guidelines.xhtml

Study Design

The study is an open randomised controlled trial (RCT) with 1:1 allocation to either a control (standard care) or an intervention group (standard care plus person-centred eHealth and telephone intervention). The trial was registered in the ClinicalTrials.gov (Identifier NCT03404583). Process evaluation and health economic analysis will also be conducted. The protocol is based on the SPIRIT (42) guidelines and the results will be reported according to established recommendations (43-45).

Study context

The study takes place in a larger city area in Western Sweden. Nine public primary health care centres are participating, all of which are located in a socioeconomically diverse area. The social insurance system in Sweden allows employees to take a maximum sick leave of 7 days without a medical certificate, and except for an initial day of "quarantine", they are financially covered by their employer during the first 14 days of illness. From day 8, a medical certificate is required that is usually obtained from a primary care or occupational health care physician. After 14 days of sick leave, benefits can be granted from the governmental Social Insurance Agency, which bases their decision upon the medical certificate.

Participants and recruitment

Recruitment of participants started in the spring of 2018 and is estimated to be completed during the fall of 2020. Patients on sick leave are consecutively screened in medical records by designated HCPs. Patients are eligible if they are on sick leave for no longer than 30 days due to a CMD that has been diagnosed by a physician. Table 1 presents the full inclusion and exclusion criteria. In the first step eligible patients receive a letter with brief information about the study.

For peer review only - http://bmjopen.bmj.com/site/about/guidelines.xhtml

Next, they are invited to make contact for more information about the study or they will be called within the upcoming week for further information. Patients willing to participate will be sent more detailed information about the study and a consent form by mail. After written consent has been received, the patient is randomised to either the control or intervention group and informed of the allocation. The randomisation is based on computer-generated random numbers and stratified by age (<50 or ≥50 years) and diagnostic group (depression, anxiety or stress reactions and disorders).

Table 1 Inclusion and exclusion criteria

**Inclusion criteria**	**Exclusion criteria**
• **Patients aged 18-65 years**<br>• **Currently employed or studying at least part-time during the past 9 months**<br>• Understand written and spoken Swedish<br>• Has a registered address<br>• **Currently on sick leave that has not exceeded 30 days, primarily due to any of the following diagnoses in ICD-10 as diagnosed by a physician: mild to moderate depression (F32 and F33), mild to moderate anxiety disorder (F41), reaction to severe stress and adjustment**	• Sick leave >14 days due to depression, anxiety disorders or stress reactions and disorders during the past 3 months<br>• Severe impairments hindering the use of the telephone and eHealth intervention<br>• Ongoing alcohol or drug abuse<br>• Any severe disease with an expected survival of <12 months or that can interfere with follow-up, or if the intervention is assessed as a burden<br>• Participating in a conflicting study

For peer review only - http://bmjopen.bmj.com/site/about/guidelines.xhtml

**disorders (F43, except post-traumatic stress disorder. F43.8A Exhaustion disorder, a diagnosis in the Swedish ICD-10, is included).**

Patient and public involvement

Public involvement is an essential part of the research conducted at GPCC. The development of the digital platform was guided by a participatory design (46). An advisory group comprising potential end-users (e.g., patient-representatives and HCPs) offered suggestions to the design of the digital platform. These end-users will be continuously consulted throughout the study period (e.g., in developing an interview guide and discussing dissemination).

Control group

Standard care commonly involves meetings with a physician to follow up on decisions regarding sick leave and RTW, as well as advice and information on self-care, but may encompass medication or cognitive behavioural therapy (10). Referral to other health care professions depends upon individual assessment and can include meetings with a physiotherapist, psychologist or psychotherapist, occupational therapist, group treatments, etc. Involving the workplace in an early, but timely stage is important regarding RTW.

Intervention group

In addition to standard care, patients allocated to the intervention group will receive PCC via telephone and a digital platform. Following allocation, patients will be e-mailed a link to create an account and access the eHealth platform. They will be contacted by an HCP to schedule a time

For peer review only - http://bmjopen.bmj.com/site/about/guidelines.xhtml

for an initial PCC conversation by telephone. This conversation, which is preferably scheduled within 1 week after initial contact, aspires to create a jointly agreed health plan, one that will serve as a foundation for future collaboration during the intervention period. A team of health care personnel with different professional backgrounds (e.g., registered nurses and physiotherapists) are involved in conducting the intervention. All received a half-day course in CMD and training in the philosophical underpinnings of PCC and how to apply these in practice. They also have a forum to meet regularly with each other and specialists in the area throughout the intervention in which they can raise questions about the intervention as well as the practice of conducting care based on person-centred ethics.

Telephone support

In the telephone conversations the patient narrative is central and the HCP will be attentive to the expressions of needs, goals, resources and experiences of the patients' current situation as well as their health status (26, 28). There is no fixed manual for how these conversations should develop nor any topics which they must cover. The most important guidance is for the HCP to be responsive to the patients' expressions of themselves as persons. The patient's narrative is documented in a health plan by either the patient or the HCP and uploaded to the eHealth platform. The health plan should capture the patients' experiences of their situation and what health-related changes they want to achieve and how, including what resources they have in terms of personal capabilities and surrounding support systems. This health plan will serve as a guide in future contacts between the HCPs and the patient. It can be modified or reformulated according to what occurs during the intervention and the patient's process. Hence, the health plan is a living document negotiated in collaboration and documented such that both the HCPs and patients (and preferably the extended network of the patients' choice) have continuous access.

For peer review only - http://bmjopen.bmj.com/site/about/guidelines.xhtml

How the telephone and eHealth support will be conducted after this first telephone call is discussed and planned in a mutual agreement between HCPs and patients.

The digital platform

The digital platform was constructed to be non-directive and create possibilities for patients to take an active part in their recovery and rehabilitation. Initially, patients and HCPs have access to the platform. Patients can also choose to expand their network by inviting people of importance to them or their rehabilitation, such as family members, other health care contacts or workplace representatives. The platform should function as a mediator through which the patients, together with their network, can monitor symptoms and be informed of their recovery process or risk of relapse or deterioration. The platform provides patients with the possibility to generate daily notes on symptoms and general wellbeing. By rating on a scale from 1-5, e.g. how well they have slept or how well they perceive their ability to concentrate, their answers will be visualised as graphs, which will provide the possibility to see trends and development over time. The patients can take private notes about their ratings and hence this may function in a diary-like manner. In addition, the patients and the HCPs can communicate with each other through messages in a chat-like forum and the platform contains an assembly of links to other web pages on CMD that the patients can use to seek information or connections. When patients invite someone to their platform page, they can decide which functions of the platform they want the invited person to access. Patients can both add new persons or delete persons that have access to the account.

Outcomes and measurements

Questionnaire data are gathered at baseline and after 3, 6, 12, 18 and 24 months from the inclusion date. Self-reported baseline characteristics of the participants' sex, age, civil status,

For peer review only - http://bmjopen.bmj.com/site/about/guidelines.xhtml

country of birth, level of education, occupation and number of working years are collected through questionnaires sent by mail. Additional baseline characteristics (e.g., income) will be gathered from the Longitudinal Integration Database for Health Insurance and Labour Market Studies (LISA).

Primary outcome

The primary outcome measure is a composite score of changes in sick leave and general self-efficacy (26, 28, 47). Patients will be classified as improved, deteriorated or unchanged according to the following standards: if the patient has a reduced sick leave percentage and an increased general self-efficacy by ≥5 units at the 6-month follow-up, the patient is classified as improved. If the patient has an increased percentage of sick leave absence or a reduced general self-efficacy by ≥5 units at the 6-month follow-up, the patient is classified as deteriorated. Those who are neither deteriorated nor improved are considered unchanged. Sick leave will be regarded from two perspectives: the duration of the initial absence and the total absence after 24 months from the inclusion date. Data on full- and part-time sick leave (25/50/75% of full-time) will be both self-reported by the participants through questionnaires and gathered from the Micro Data for the Analysis of Social Insurance register (MiDAS). Self-efficacy will be measured using the General Self-Efficacy Scale (GSE) (48, 49), a 10-item self-assessment questionnaire measuring a person's general sense of competence towards dealing with unforeseen situations. Responses are made on a four-point scale (1 = not at all true, 2 = hardly true, 3 = moderately true, 4 = exactly true), with a composite score based on the sum of all items. The total score ranges from 10 to 40, with higher scores indicating a higher sense of self-efficacy.

Secondary outcomes

For peer review only - http://bmjopen.bmj.com/site/about/guidelines.xhtml

Additional analyses will be conducted and based on data from the following instruments:

- EuroQol 5-Dimension health state questionnaire (EQ-5D) (50)
- Shirom-Melamed Burnout Questionnaire (SMBQ) (51)
- Perceived Stress Scale (PSS) (52)
- Self-rated Exhaustion Disorder (s-ED) (53)
- Hospital Anxiety and Depression Scale (HADS) (54)
- Multidimensional Fatigue Symptom Inventory (MFI-20) (55)
- Self-Efficacy for Managing Chronic Disease 6-item Scale (SEMCD) (56)
- Sheehan Disability Scale (SDS) (57)

Power/Sample size

To achieve a power of 80% based on a one-sided alpha error of 0.05 91 participants would be needed in each study group to detect an improvement in the composite score (20% in the control group vs. 40% in the intervention group). We aim to include a minimum of 110 patients per group to have some margin for dropouts or withdrawals.

Data Analysis

Descriptive and analytic statistics will be conducted to compare the study groups. Logistic regression will be used on the primary outcome measure to calculate odds ratios with 95% confidence intervals (CIs). For highly skewed data (e.g., cost data), a bootstrap method will be used to estimate CIs (58). Parametric and nonparametric statistics will be carried out for

For peer review only

For peer review only - http://bmjopen.bmj.com/site/about/guidelines.xhtml

secondary outcome measures. Intention-to-treat and, if needed, per-protocol analyses will be performed. Subgroup analyses will be conducted on, e.g., sex, age and diagnostic groups.

Economic evaluation

The economic evaluation will assess whether the cost of introducing this add-on PCC intervention is acceptable compared with the achieved health effects e.g., measured as HRQoL collected through questionnaires. Missing questionnaire data will be assumed missing-at-random and thus handled using multiple imputations. The health state index derived from the EQ-5D questionnaire will be translated into quality-adjusted life-years using an area under the curve calculation and the Swedish experience-based valuation (59) and, as a sensitivity analysis, the society-based valuation for the UK (60). The calculation of costs from a societal perspective will include costs for prescribed drugs, health care encounters and lost productivity. Drug costs will be obtained from the Swedish Prescribed Drug Register, held by the National Board of Health and Welfare. Health care encounters will be obtained from the regional patient register VEGA and converted to costs using diagnosis-related group (DRG) weights (available for all encounters in specialised health care) and the national cost per one DRG, whereas unit costs presented in national statistics will be used for primary care contacts (since DRGs are not available for primary care). In addition, we will collect information about the time spent by HCPs interacting with participants on the platform and by phone. Lost productivity will be obtained from both questionnaires and the MiDAS register, under the authority of the Social Insurance Agency, to ensure all absenteeism is recorded. This is done because the national register only fully covers days off from work that are reimbursed by the agency. Indirect costs for lost productivity will then be calculated using "the human capital approach", which includes multiplying time off from work by mean wages (by age groups) and the social security contribution paid by employers (61).

For peer review only - http://bmjopen.bmj.com/site/about/guidelines.xhtml

Total costs and costs per payer category (patients, market sector, health care providers/regions and other authorities/tax-based funds) will be reported. The incremental cost will then be compared with incremental health effects using the incremental cost-effectiveness ratio (ICER). To explore the value of the intervention the ICER will be compared with the informal cost-effectiveness threshold used in Sweden of SEK 500,000 and to alternative willingness-to-pay thresholds using the cost-effectiveness acceptability curve.

Process evaluation

To link the outcomes of the intervention to the implementation process we will explore three complementary aspects of the intervention: 1) how much and in what way participants have used the PCC intervention, 2) how they experienced it and 3) whether it has been used as intended (62). Quantitative data will be collected from survey questions on the use and ratings of telephone and platform support, which will be attached to the 3- and 6-month questionnaires sent to those in the intervention group. We will also collect data on the use of different functions of the intervention and number and modes of contact between HCPs, patients and persons invited to the platform during the intervention period. Qualitative data will consist of transcribed audio recordings of telephone conversations between HCPs and patients. In addition, open questions in the 3-and 6-month surveys permit elaboration on experiences of the activities and delivery of the intervention. We will also interview a sample of patients in the intervention group, as well as involved HCPs and persons invited to the platform for an in-depth exploration of their experiences of the intervention. Qualitative data will be analysed using qualitative analysis methods and quantitative data using descriptive statistics. Using both quantitative and qualitative data allows applying mixed-methods analysis to triangulate the data, which increases the reliability of the study outcome.

For peer review only - http://bmjopen.bmj.com/site/about/guidelines.xhtml

Ethics and dissemination

The study was approved by the Regional Ethical Review Board in Gothenburg, Sweden (DNr 497-17, T 023-18, T 526-18). All participants agreed to participate and signed a written consent form after receiving written and oral information about the study. Informed, written consent will also be asked of participants accepting to be interviewed. Studies in the project will be published in peer-reviewed scientific journals and presented at national and international conferences. This project is part of a research programme at the GPCC, where extensive work is ongoing to disseminate knowledge on and implementation of PCC.

Discussion

The person-centred health care process is based on a continuous negotiation to understand the general and unique premises of each patient's situation, with decisions and actions guided by that understanding (30, 63). Consequently, such care varies between patients, which explains the complexity of PCC and person-centred interventions as they are individually tailored rather than standardised. In this study we have chosen to accompany the RCT with a process evaluation to understand more about how participants interact with the intervention's components, which is essential to understand how a complex intervention works (62). Hawe et al (64) suggest that process evaluation of complex interventions needs to clearly state what is assumed to be the essential element of change. As long as the essential elements are not altered, other intervention components can be adapted to serve the local context. Applied to the present study, we assume that the essential element to generate change is the quality of the person-centredness of communication. Considering the population of primary care patients with a CMD on sick leave,

For peer review only

part of the recent increase in mental illness-related sick leave is possibly explained by both work-life demands and stressors in private life (65). Moreover, psychosocial stress exposure can be conceptualised as an imbalance between demands, support and resources such as control (66). Consequently, we believe that it is essential with a person-centred communication rooted in an understanding of the patient as both vulnerable and capable and that reaching agreements on care and rehabilitation build upon the knowledge of the patient's circumstances, needs, wishes and resources. However, we see no need to be prescriptive regarding how the participants should use the different features of the intervention. For instance, this could mean that some patients won't use the platform at all, whereas others will prefer communication via the platform over telephone conversations. How the intervention is finally executed will be clarified in the process evaluation. We included self-efficacy as a primary outcome measure for the RCT. This choice is based mainly on two reasons. First, several studies have suggested self-efficacy as an important psychological resource in an RTW process (20-22). Second, studies on PCC interventions have reported positive effects of self-efficacy (26-28, 38). However, how this effect is achieved is still unclear and hence we intend to further explore the connection between PCC and self-efficacy as part of the process evaluation.

The study design has some limitations. Although the study is conducted in cooperation with primary care centres, the intervention is mainly performed by designated HCPs situated in a research centre in a separate location. One advantage of that arrangement is the possibility to follow the process closely thanks to full access to all patient-staff communication. However, the study results will have to be considered in light of that and future adaptions may be needed before implementing the intervention in other contexts and settings. Another limitation is the language restriction. It has not been feasible to involve interpreters and therefore only patients who can

For peer review only

independently manage to communicate in Swedish will be included in the study. Participation also require access to an internet device.

The present study corresponds to the current call for interventions targeting sick leave in CMD patients. The design of the intervention is consistent with what previous reviews have shown promising, e.g., facilitating collaboration, using multiple components (19) and providing possibilities of involving the workplace (15-19). The choice to develop and evaluate an eHealth intervention was largely because of the ambition to limit the number of physical appointments the patients would need to attend and reach the patients in their everyday life at home. We believe this represents one of the potential benefits of eHealth services. However, it is fundamental that also remotely delivered health care is person-centred. We also believe that developing interventions, which are not limited to a certain professional category and targeting not only one diagnosis, is necessary considering the current pressure on first-line health care facilities for CMDs. We consider it a strength that our study investigates PCC as an addition to, not in comparison with, standard care. This intervention approach is expected to gain new knowledge on how PCC can contribute to the challenges facing healthcare systems with a large number of persons seeking help for their mental illness and the difficulties in coping with work and everyday life.

References

1. OECD. Mental Health and Work: Sweden. Paris: OECD Publishing; 2013.
2. Henderson M, Glozier N, Elliott KH. Long term sickness absence. BMJ. 2005;330(7495):802-3.
3. Henderson M, Harvey SB, Øverland S, Mykletun A, Hotopf M. Work and common psychiatric disorders. London, England2011. p. 198-207.

For peer review only - http://bmjopen.bmj.com/site/about/guidelines.xhtml

4. Försäkringskassan [Swedish Social Insurance Agency]. Social Insurance in Figures 2019. https://www.forsakringskassan.se/wps/wcm/connect/cec4cea8-1d6c-4895-b442-bc3b64735b09/socialforsakringen-i-siffror-2019-engelsk.pdf?MOD=AJPERES&CVID=2019.
5. Försäkringskassan [Swedish Social Insurance Agency]. Uppföljning av sjukfrånvarons utveckling 2019. https://www.forsakringskassan.se/wps/wcm/connect/fb32bcf5-7ed4-4356-b18b-6adc7ca20fcd/1573-19-svar-regeringsuppdrag.pdf?MOD=AJPERES&CVID=2019.
6. Lidwall U. Sick leave diagnoses and return to work: a Swedish register study. Disability and Rehabilitation. 2015;37(5):396-410.
7. Försäkringskassan [Swedish Social Insurance Agency]. Sjukfrånvarons utveckling 2017. https://www.forsakringskassan.se/wps/wcm/connect/1596d32b-7ff7-4811-8215-d90cb9c2f38d/socialforsakringsrapport-2017-13.pdf?MOD=AJPERES&CVID=2017.
8. Dewa CS, Loong D, Bonato S. Work outcomes of sickness absence related to mental disorders: A systematic literature review. BMJ Open. 2014;4(7).
9. Sundquist J, Ohlsson H, Sundquist K, Kendler KS. Common adult psychiatric disorders in Swedish primary care where most mental health patients are treated. BMC Psychiatry. 2017;17(1):235.
10. Socialstyrelsen [National Board of Health and Welfare]. Nationella riktlinjer för vård vid depression och ångestsyndrom. Socialstyrelsen; 2017.
11. Soegaard H. Variation in effect of intervention studies in research on sickness absence. Open Access Journal of Clinical Trials. 2012;4:1-20.
12. Nieuwenhuijsen K, Bültmann U, Neumeyer-Gromen A, Verhoeven AC, Verbeek JH, Feltz-Cornelis CM. Interventions to improve occupational health in depressed people. Cochrane Database of Systematic Reviews. 2008(2).
13. van der Klink JJL, Blonk RWB, Schene AH, van Dijk FJH. Reducing long term sickness absence by an activating intervention in adjustment disorders: a cluster randomised controlled design. Occupational and Environmental Medicine. 2003;60(6):429.
14. Blank L, Peters J, Pickvance S, Wilford J, MacDonald E. A Systematic Review of the Factors which Predict Return to Work for People Suffering Episodes of Poor Mental Health. Journal of Occupational Rehabilitation. 2008;18(1):27-34.
15. Vogel N, Schandelmaier S, Zumbrunn T, Ebrahim S, de Boer WE, Busse JW, et al. Return-to-work coordination programmes for improving return to work in workers on sick leave. Cochrane Database Syst Rev. 2017;3:Cd011618.
16. van Vilsteren M, van Oostrom SH, de Vet HC, Franche RL, Boot CR, Anema JR. Workplace interventions to prevent work disability in workers on sick leave. Cochrane Database Syst Rev. 2015(10):Cd006955.
17. Arends I, Bruinvels DJ, Rebergen DS, Nieuwenhuijsen K, Madan I, Neumeyer-Gromen A, et al. Interventions to facilitate return to work in adults with adjustment disorders. Cochrane Database Syst Rev. 2012;12:Cd006389.
18. Nieuwenhuijsen K, Faber B, Verbeek JH, Neumeyer-Gromen A, Hees HL, Verhoeven AC, et al. Interventions to improve return to work in depressed people. Cochrane Database Syst Rev. 2014(12):Cd006237.
19. Mikkelsen MB, Rosholm M. Systematic review and meta-analysis of interventions aimed at enhancing return to work for sick-listed workers with common mental disorders, stress-related disorders, somatoform disorders and personality disorders. Occup Environ Med. 2018;75(9):675-86.

For peer review only - http://bmjopen.bmj.com/site/about/guidelines.xhtml

20. Etuknwa A, Daniels K, Eib C. Sustainable Return to Work: A Systematic Review Focusing on Personal and Social Factors. Journal of Occupational Rehabilitation. 2019.
21. Volker D, Zijlstra-Vlasveld MC, Brouwers EP, van Lomwel AG, van der Feltz-Cornelis CM. Return-to-Work Self-Efficacy and Actual Return to Work Among Long-Term Sick-Listed Employees. J Occup Rehabil. 2015;25(2):423-31.
22. Nigatu Y, Liu Y, Uppal M, McKinney S, Gillis K, Rao S, et al. Prognostic factors for return to work of employees with common mental disorders: a meta-analysis of cohort studies. Social Psychiatry and Psychiatric Epidemiology. 2017;52(10):1205-15.
23. Bandura A. Social cognitive theory: an agentic perspective. Annu Rev Psychol. 2001;52:1-26.
24. Bandura A. Self-efficacy: toward a unifying theory of behavioral change. Psychol Rev. 1977;84(2):191-215.
25. Seggelen - Damen Iv, Dam Kv. Self-reflection as a mediator between self-efficacy and well-being. Journal of Managerial Psychology. 2016;31(1):18-33.
26. Fors A, Ekman I, Taft C, Bjorkelund C, Frid K, Larsson ME, et al. Person-centred care after acute coronary syndrome, from hospital to primary care - A randomised controlled trial. Int J Cardiol. 2015;187:693-9.
27. Fors A, Taft C, Ulin K, Ekman I. Person-centred care improves self-efficacy to control symptoms after acute coronary syndrome: a randomized controlled trial. European Journal of Cardiovascular Nursing. 2016;15(2):186-94.
28. Fors A, Blanck E, Ali L, Ekberg-Jansson A, Fu M, Kjellberg IL, et al. Effects of a person-centred telephone-support in patients with chronic obstructive pulmonary disease and/or chronic heart failure - A randomized controlled trial. Plos One. 2018;13(8).
29. Ekman I, Swedberg K, Taft C, Lindseth A, Norberg A, Brink E, et al. Person-centered care--ready for prime time. Eur J Cardiovasc Nurs. 2011;10(4):248-51.
30. Entwistle VA, Watt IS. Treating Patients as Persons: A Capabilities Approach to Support Delivery of Person-Centered Care. The American Journal of Bioethics. 2013;13(8):29-39.
31. Leplege A, Gzil F, Cammelli M, Lefeve C, Pachoud B, Ville I. Person-centredness: conceptual and historical perspectives. Disability and rehabilitation. 2007;29(20-21):1555.
32. Mccormack B, Mccance, T Personhood and Person-Centredness. Person-Centred Nursing. Oxford, UK: Wiley-Blackwell; 2010. p. 5-20.
33. Brannstrom M, Boman K. Effects of person-centred and integrated chronic heart failure and palliative home care. PREFER: a randomized controlled study. Eur J Heart Fail. 2014;16(10):1142-51.
34. Wolf A, Vella R, Fors A. The impact of person-centred care on patients' care experiences in relation to educational level after acute coronary syndrome: secondary outcome analysis of a randomised controlled trial. Eur J Cardiovasc Nurs. 2019;18(4):299-308.
35. Dudas K, Olsson LE, Wolf A, Swedberg K, Taft C, Schaufelberger M, et al. Uncertainty in illness among patients with chronic heart failure is less in person-centred care than in usual care. Eur J Cardiovasc Nurs. 2013;12(6):521-8.
36. Hansson E, Ekman I, Swedberg K, Wolf A, Dudas K, Ehlers L, et al. Person-centred care for patients with chronic heart failure - a cost-utility analysis. Eur J Cardiovasc Nurs. 2016;15(4):276-84.
37. Gyllensten H, Koinberg I, Carlstrom E, Olsson LE, Hansson Olofsson E. Economic evaluation of a person-centred care intervention in head and neck oncology: results from a randomized controlled trial. Support Care Cancer. 2019;27(5):1825-34.

For peer review only

38. Wolf A, Fors A, Ulin K, Thorn J, Swedberg K, Ekman I. An eHealth Diary and Symptom-Tracking Tool Combined With Person-Centered Care for Improving Self-Efficacy After a Diagnosis of Acute Coronary Syndrome: A Substudy of a Randomized Controlled Trial. J Med Internet Res. 2016;18(2):e40.
39. Heckemann B, Wolf A, Ali L, Sonntag SM, Ekman I. Discovering untapped relationship potential with patients in telehealth: a qualitative interview study. BMJ Open. 2016;6(3):e009750.
40. Eysenbach G. What is e-health? J Med Internet Res. 2001;3(2):e20.
41. Andersson G. Internet-Delivered Psychological Treatments. Annual Review of Clinical Psychology. 2016;12(1):157-79.
42. Chan A-W, Tetzlaff JM, Gøtzsche PC, Altman DG, Mann H, Berlin JA, et al. SPIRIT 2013 explanation and elaboration: guidance for protocols of clinical trials. BMJ : British Medical Journal. 2013;346:e7586.
43. Moher D, Schulz KF, Altman DG. The CONSORT statement: revised recommendations for improving the quality of reports of parallel-group randomised trials. Lancet. 2001;357(9263):1191-4.
44. Husereau D, Drummond M, Petrou S, Carswell C, Moher D, Greenberg D, et al. Consolidated Health Economic Evaluation Reporting Standards (CHEERS) statement. BMJ : British Medical Journal. 2013;346:f1049.
45. Pinnock H, Barwick M, Carpenter CR, Eldridge S, Grandes G, Griffiths CJ, et al. Standards for Reporting Implementation Studies (StaRI): explanation and elaboration document. BMJ Open. 2017;7(4):e013318.
46. Sanders EBN, Stappers PJ. Co-creation and the new landscapes of design. CoDesign. 2008;4(1):5-18.
47. Packer M. Proposal for a new clinical end point to evaluate the efficacy of drugs and devices in the treatment of chronic heart failure. J Card Fail. 2001;7(2):176-82.
48. Schwarzer R, & Jerusalem, M. . Generalized Self-Efficacy scale. In: Johnston M, Wright, S., Weinman, J. , editor. Measures in health psychology: A user's portfolio. Windsor: UK: NFER-NELSON; 1995. p. 35-7.
49. Love J, Moore CD, Hensing G. Validation of the Swedish translation of the General Self-Efficacy scale. Qual Life Res. 2012;21(7):1249-53.
50. Brooks R. EuroQol: the current state of play. Health policy. 1996;37(1):53-72.
51. Lundgren-Nilsson A, Jonsdottir IH, Pallant J, Ahlborg G, Jr. Internal construct validity of the Shirom-Melamed Burnout Questionnaire (SMBQ). BMC Public Health. 2012;12:1.
52. Cohen S, Kamarck T, Mermelstein R. A global measure of perceived stress. J Health Soc Behav. 1983;24(4):385-96.
53. Glise K, Hadzibajramovic E, Jonsdottir IH, Ahlborg G, Jr. Self-reported exhaustion: a possible indicator of reduced work ability and increased risk of sickness absence among human service workers. Int Arch Occup Environ Health. 2010;83(5):511-20.
54. Zigmond AS, Snaith RP. The hospital anxiety and depression scale. Acta Psychiatr Scand. 1983;67(6):361-70.
55. Smets EM, Garssen B, Bonke B, De Haes JC. The Multidimensional Fatigue Inventory (MFI) psychometric qualities of an instrument to assess fatigue. J Psychosom Res. 1995;39(3):315-25.
56. Lorig KR, Sobel DS, Ritter PL, Laurent D, Hobbs M. Effect of a self-management program on patients with chronic disease. Eff Clin Pract. 2001;4(6):256-62.

For peer review only

57. Leon AC, Olfson M, Portera L, Farber L, Sheehan DV. Assessing psychiatric impairment in primary care with the Sheehan Disability Scale. Int J Psychiatry Med. 1997;27(2):93-105.
58. Barber JA, Thompson SG. Analysis of cost data in randomized trials: an application of the non-parametric bootstrap. Statistics in Medicine. 2000;19(23):3219-36.
59. Burström K, Sun S, Gerdtham U-G, Henriksson M, Johannesson M, Levin L-Å, et al. Swedish experience-based value sets for EQ-5D health states. Quality of Life Research. 2014;23(2):431-42.
60. Dolan P, Gudex C, Kind P, Williams A. A social tariff for EuroQol: results from a UK general population survey. 1995.
61. Krol M, Brouwer W, Rutten F. Productivity Costs in Economic Evaluations: Past, Present, Future. PharmacoEconomics. 2013;31(7):537-49.
62. Moore GF, Audrey S, Barker M, Bond L, Bonell C, Hardeman W, et al. Process evaluation of complex interventions: Medical Research Council guidance. BMJ : British Medical Journal. 2015;350:h1258.
63. McCormack B, McCance T. Person-centred practice in nursing and health care : theory and practice. Second edition ed: Chichester : Wiley Blackwell; 2017.
64. Hawe P, Shiell A, Riley T. Complex interventions: how "out of control" can a randomised controlled trial be? BMJ. 2004;328(7455):1561-3.
65. Hasselberg K, Jonsdottir IH, Ellbin S, Skagert K. Self-reported stressors among patients with exhaustion disorder: an exploratory study of patient records. BMC psychiatry. 2014;14(1):66.
66. Karasek R. Healthy work : stress, productivity, and the reconstruction of working life. Theorell T, editor. New York, N.Y.: New York, N.Y. : Basic Books; 1990.

Author Contributions

All authors were involved in the design of the study. MC drafted the manuscript with critical input from LA, IE, KG, IJ, HG, KS and AF. AF is the principal investigator and grant holder of the investigation. All authors reviewed, edited and approved the final version of the manuscript.

Funding statement

This work was supported by The Swedish Research Council for Health, Working Life and Welfare (reference number 2016-07418, 2017-00557 and 2019-01726). The funder has no role in the design of the study, data collection, analysis, or interpretation. The study was financed by grants from the

For peer review only - http://bmjopen.bmj.com/site/about/guidelines.xhtml

Swedish state under the agreement between the Swedish government and the country councils, the ALF agreement (ALFGBG-772191 and ALFGBG-932659).

Competing interests' statement

Nothing to declare

Word count

3928

For peer review only

Reporting checklist for protocol of a clinical trial.

Based on the SPIRIT guidelines.

Instructions to authors

Complete this checklist by entering the page numbers from your manuscript where readers will find each of the items listed below.

Your article may not currently address all the items on the checklist. Please modify your text to include the missing information. If you are certain that an item does not apply, please write "n/a" and provide a short explanation.

Upload your completed checklist as an extra file when you submit to a journal.

In your methods section, say that you used the SPIRITreporting guidelines, and cite them as:

Chan A-W, Tetzlaff JM, Altman DG, Laupacis A, Gøtzsche PC, Krleža-Jerić K, Hróbjartsson A, Mann H, Dickersin K, Berlin J, Doré C, Parulekar W, Summerskill W, Groves T, Schulz K, Sox H, Rockhold FW, Rennie D, Moher D. SPIRIT 2013 Statement: Defining standard protocol items for clinical trials. Ann Intern Med. 2013;158(3):200-207

		Reporting Item	Page Number
**Administrative information**			
Title	#1	Descriptive title identifying the study design, population, interventions, and, if applicable, trial acronym	1
Trial registration	#2a	Trial identifier and registry name. If not yet registered, name of intended registry	6
Trial registration: data set	#2b	All items from the World Health Organization Trial Registration Data Set	n/a
Protocol version	#3	Date and version identifier	n/a
Funding	#4	Sources and types of financial, material, and other support	21-22
Roles and responsibilities: contributorship	#5a	Names, affiliations, and roles of protocol contributors	21

For peer review only

Roles and responsibilities: sponsor contact information	#5b	Name and contact information for the trial sponsor	n/a
Roles and responsibilities: sponsor and funder	#5c	Role of study sponsor and funders, if any, in study design; collection, management, analysis, and interpretation of data; writing of the report; and the decision to submit the report for publication, including whether they will have ultimate authority over any of these activities	21-22
Roles and responsibilities: committees	#5d	Composition, roles, and responsibilities of the coordinating centre, steering committee, endpoint adjudication committee, data management team, and other individuals or groups overseeing the trial, if applicable (see Item 21a for data monitoring committee)	n/a
**Introduction**			
Background and rationale	#6a	Description of research question and justification for undertaking the trial, including summary of relevant studies (published and unpublished) examining benefits and harms for each intervention	3-5
Background and rationale: choice of comparators	#6b	Explanation for choice of comparators	8
Objectives	#7	Specific objectives or hypotheses	5
Trial design	#8	Description of trial design including type of trial (eg, parallel group, crossover, factorial, single group), allocation ratio, and framework (eg, superiority, equivalence, non-inferiority, exploratory)	6
**Methods: Participants, interventions, and outcomes**			
Study setting	#9	Description of study settings (eg, community clinic, academic hospital) and list of countries where data will be collected. Reference to where list of study sites can be obtained	6
Eligibility criteria	#10	Inclusion and exclusion criteria for participants. If applicable, eligibility criteria for study centres and individuals who will	6-7

For peer review only

		perform the interventions (eg, surgeons, psychotherapists)	
Interventions: description	#11a	Interventions for each group with sufficient detail to allow replication, including how and when they will be administered	8-10
Interventions: modifications	#11b	Criteria for discontinuing or modifying allocated interventions for a given trial participant (eg, drug dose change in response to harms, participant request, or improving / worsening disease)	n/a
Interventions: adherance	#11c	Strategies to improve adherence to intervention protocols, and any procedures for monitoring adherence (eg, drug tablet return; laboratory tests)	n/a
Interventions: concomitant care	#11d	Relevant concomitant care and interventions that are permitted or prohibited during the trial	n/a
Outcomes	#12	Primary, secondary, and other outcomes, including the specific measurement variable (eg, systolic blood pressure), analysis metric (eg, change from baseline, final value, time to event), method of aggregation (eg, median, proportion), and time point for each outcome. Explanation of the clinical relevance of chosen efficacy and harm outcomes is strongly recommended	10-12
Participant timeline	#13	Time schedule of enrolment, interventions (including any run-ins and washouts), assessments, and visits for participants. A schematic diagram is highly recommended (see Figure)	6
Sample size	#14	Estimated number of participants needed to achieve study objectives and how it was determined, including clinical and statistical assumptions supporting any sample size calculations	12
Recruitment	#15	Strategies for achieving adequate participant enrolment to reach target sample size	n/a
**Methods: Assignment of interventions (for controlled trials)**			
Allocation: sequence generation	#16a	Method of generating the allocation sequence (eg, computer-generated random numbers), and list of any factors for stratification. To reduce predictability of a random sequence, details of any planned restriction (eg, blocking) should be provided in a separate document that is unavailable to those who enrol participants or assign interventions	7

For peer review only

Allocation concealment mechanism	#16b	Mechanism of implementing the allocation sequence (eg, central telephone; sequentially numbered, opaque, sealed envelopes), describing any steps to conceal the sequence until interventions are assigned	n/a
Allocation: implementation	#16c	Who will generate the allocation sequence, who will enrol participants, and who will assign participants to interventions	6
Blinding (masking)	#17a	Who will be blinded after assignment to interventions (eg, trial participants, care providers, outcome assessors, data analysts), and how	n/a
Blinding (masking): emergency unblinding	#17b	If blinded, circumstances under which unblinding is permissible, and procedure for revealing a participant's allocated intervention during the trial	n/a
**Methods: Data collection, management, and analysis**			
Data collection plan	#18a	Plans for assessment and collection of outcome, baseline, and other trial data, including any related processes to promote data quality (eg, duplicate measurements, training of assessors) and a description of study instruments (eg, questionnaires, laboratory tests) along with their reliability and validity, if known. Reference to where data collection forms can be found, if not in the protocol	10-14
Data collection plan: retention	#18b	Plans to promote participant retention and complete follow-up, including list of any outcome data to be collected for participants who discontinue or deviate from intervention protocols	n/a
Data management	#19	Plans for data entry, coding, security, and storage, including any related processes to promote data quality (eg, double data entry; range checks for data values). Reference to where details of data management procedures can be found, if not in the protocol	n/a
Statistics: outcomes	#20a	Statistical methods for analysing primary and secondary outcomes. Reference to where other details of the statistical analysis plan can be found, if not in the protocol	12-14
Statistics: additional analyses	#20b	Methods for any additional analyses (eg, subgroup and adjusted analyses)	12-14

For peer review only

Statistics: analysis population and missing data	#20c	Definition of analysis population relating to protocol non-adherence (eg, as randomised analysis), and any statistical methods to handle missing data (eg, multiple imputation)	13
**Methods: Monitoring**			
Data monitoring: formal committee	#21a	Composition of data monitoring committee (DMC); summary of its role and reporting structure; statement of whether it is independent from the sponsor and competing interests; and reference to where further details about its charter can be found, if not in the protocol. Alternatively, an explanation of why a DMC is not needed	n/a
Data monitoring: interim analysis	#21b	Description of any interim analyses and stopping guidelines, including who will have access to these interim results and make the final decision to terminate the trial	n/a
Harms	#22	Plans for collecting, assessing, reporting, and managing solicited and spontaneously reported adverse events and other unintended effects of trial interventions or trial conduct	n/a
Auditing	#23	Frequency and procedures for auditing trial conduct, if any, and whether the process will be independent from investigators and the sponsor	n/a
**Ethics and dissemination**			
Research ethics approval	#24	Plans for seeking research ethics committee / institutional review board (REC / IRB) approval	15
Protocol amendments	#25	Plans for communicating important protocol modifications (eg, changes to eligibility criteria, outcomes, analyses) to relevant parties (eg, investigators, REC / IRBs, trial participants, trial registries, journals, regulators)	n/a
Consent or assent	#26a	Who will obtain informed consent or assent from potential trial participants or authorised surrogates, and how (see Item 32)	7, 15
Consent or assent: ancillary studies	#26b	Additional consent provisions for collection and use of participant data and biological specimens in ancillary studies, if applicable	n/a
Confidentiality	#27	How personal information about potential and enrolled participants will be collected, shared, and maintained in order to protect confidentiality before, during, and after the trial	15

For peer review only

Declaration of interests	#28	Financial and other competing interests for principal investigators for the overall trial and each study site	22
Data access	#29	Statement of who will have access to the final trial dataset, and disclosure of contractual agreements that limit such access for investigators	n/a
Ancillary and post trial care	#30	Provisions, if any, for ancillary and post-trial care, and for compensation to those who suffer harm from trial participation	n/a
Dissemination policy: trial results	#31a	Plans for investigators and sponsor to communicate trial results to participants, healthcare professionals, the public, and other relevant groups (eg, via publication, reporting in results databases, or other data sharing arrangements), including any publication restrictions	15
Dissemination policy: authorship	#31b	Authorship eligibility guidelines and any intended use of professional writers	n/a
Dissemination policy: reproducible research	#31c	Plans, if any, for granting public access to the full protocol, participant-level dataset, and statistical code	n/a
**Appendices**			
Informed consent materials	#32	Model consent form and other related documentation given to participants and authorised surrogates	n/a
Biological specimens	#33	Plans for collection, laboratory evaluation, and storage of biological specimens for genetic or molecular analysis in the current trial and for future use in ancillary studies, if applicable	n/a

The SPIRIT checklist is distributed under the terms of the Creative Commons Attribution License CC-BY-ND 3.0. This checklist was completed on 05. February 2020 using https://www.goodreports.org/, a tool made by the EQUATOR Network in collaboration with Penelope.ai

For peer review only

BMJ Open

A person-centred eHealth intervention for patients on sick leave due to common mental disorders: Protocol of a randomised controlled trial and process evaluation (PROMISE)

Journal:	*BMJ Open*
Manuscript ID	bmjopen-2020-037515.R1
Article Type:	Protocol
Date Submitted by the Author:	27-May-2020
Complete List of Authors:	Cederberg, Matilda; University of Gothenburg Institute of Health and Care Sciences, Centre for Person-Centred Care, Sahlgrenska Academy<br>Ali, Lilas; University of Gothenburg Institute of Health and Care Sciences, Centre for Person-Centred Care, Sahlgrenska Academy; Sahlgrenska University Hospital, Department of Psychiatry<br>Ekman, Inger; University of Gothenburg Institute of Health and Care Sciences, Centre for Person-Centred Care, Sahlgrenska Academy; Sahlgrenska University Hospital, Department of Internal medicine and geriatrics<br>Glise, Kristina; Institute of Stress Medicine<br>Jonsdottir, Ingibjörg; Institute of Stress Medicine; University of Gothenburg Sahlgrenska Academy, School of Public Health and Community medicine<br>Gyllensten, Hanna; University of Gothenburg Institute of Health and Care Sciences, Centre for Person-Centred Care, Sahlgrenska Academy<br>Swedberg, Karl; University of Gothenburg Institute of Health and Care Sciences, Centre for Person-Centred Care, Sahlgrenska Academy; University of Gothenburg Sahlgrenska Academy, Department of Molecular and Clinical Medicine<br>Fors, Andreas; University of Gothenburg Institute of Health and Care Sciences, Centre for Person-Centred Care, Sahlgrenska Academy; Närhälsan Research and Development Primary Health Care
**Primary Subject Heading**:	Mental health
Secondary Subject Heading:	Public health, Patient-centred medicine
Keywords:	MENTAL HEALTH, PRIMARY CARE, PUBLIC HEALTH, Information technology < BIOTECHNOLOGY & BIOINFORMATICS, Quality in health care < HEALTH SERVICES ADMINISTRATION & MANAGEMENT

SCHOLARONE™
Manuscripts

I, the Submitting Author has the right to grant and does grant on behalf of all authors of the Work (as defined in the below author licence), an exclusive licence and/or a non-exclusive licence for contributions from authors who are: i) UK Crown employees; ii) where BMJ has agreed a CC-BY licence shall apply, and/or iii) in accordance with the terms applicable for US Federal Government officers or employees acting as part of their official duties; on a worldwide, perpetual, irrevocable, royalty-free basis to BMJ Publishing Group Ltd ("BMJ") its licensees and where the relevant Journal is co-owned by BMJ to the co-owners of the Journal, to publish the Work in this journal and any other BMJ products and to exploit all rights, as set out in our licence.

The Submitting Author accepts and understands that any supply made under these terms is made by BMJ to the Submitting Author unless you are acting as an employee on behalf of your employer or a postgraduate student of an affiliated institution which is paying any applicable article publishing charge ("APC") for Open Access articles. Where the Submitting Author wishes to make the Work available on an Open Access basis (and intends to pay the relevant APC), the terms of reuse of such Open Access shall be governed by a Creative Commons licence – details of these licences and which Creative Commons licence will apply to this Work are set out in our licence referred to above.

Other than as permitted in any relevant BMJ Author's Self Archiving Policies, I confirm this Work has not been accepted for publication elsewhere, is not being considered for publication elsewhere and does not duplicate material already published. I confirm all authors consent to publication of this Work and authorise the granting of this licence.

For peer review only

A person-centred eHealth intervention for patients on sick leave due to common mental disorders: Protocol of a randomised controlled trial and process evaluation (PROMISE).

Matilda Cederberg[1,2], Lilas Ali[1,2,3], Inger Ekman[1,2,4], Kristina Glise[5], Ingibjörg H Jonsdottir[5,6] Hanna Gyllensten[1,2], Karl Swedberg[1,2,4,7], Andreas Fors[1,2,8]

1 Institute of Health and Care Sciences, Sahlgrenska Academy, University of Gothenburg, Gothenburg, Sweden.
2 Centre for Person-Centred Care (GPCC), University of Gothenburg, Gothenburg, Sweden.
3 Sahlgrenska University Hospital, Psychiatric department, Gothenburg, Sweden.
4 Sahlgrenska university hospital/Östra/Department of Internal medicine and geriatrics, Gothenburg, Sweden.
5 The Institute of Stress Medicine, Region Västra Götaland, Gothenburg, Sweden
6 School of Public Health and Community Medicine, Sahlgrenska Academy, University of Gothenburg, Gothenburg, Sweden
7 Department of Molecular and Clinical Medicine, Sahlgrenska Academy, University of Gothenburg, Gothenburg, Sweden.
8 Närhälsan Research and Development Primary Health Care, Region Västra Götaland, Sweden.

Corresponding author: Matilda Cederberg, Institute of Health Care Sciences, Centre for Person-Centred Care, Sahlgrenska Academy, Box 457, 405 30 Gothenburg, Sweden. matilda.cederberg@gu.se

For peer review only

Abstract

Introduction The number of people dealing with common mental disorders (CMDs) is a major concern in many countries, including Sweden. Sickness absence resulting from CMDs is often long-lasting and advancing return to work is a complex process impacted by several factors, among which self-efficacy appears to be an important personal resource. Developing health care strategies that build on person-centred care (PCC) is needed to advance recovery through collaboration and enhanced self-efficacy.

Methods and analysis This study is an open randomised controlled trial comparing a control group receiving standard care with an intervention group receiving standard care plus person-

For peer review only - http://bmjopen.bmj.com/site/about/guidelines.xhtml

centred care by telephone and a digital platform. The primary outcome measure is a composite score of changes in sick leave and self-efficacy. Participants will include 220 primary care patients on sick leave due to CMDs and data will mainly be collected through questionnaires at baseline and 3, 6, 12 and 24 months from the inclusion date. Inclusion is ongoing and expected to be completed during the fall of 2020. A process and health economic evaluation will also be conducted.

Ethics and dissemination This study was approved by the Regional Ethical Review Board in Gothenburg, Sweden. Results will be published in peer-reviewed scientific journals and presented at national and international scientific conferences. This project is part of a broader research programme conducted at the Gothenburg Centre for Person-Centred Care, where extensive work is undertaken to disseminate knowledge on and implementation of PCC.

For peer review only

Article Summary

Strengths and limitations of this study

- The intervention contains multiple components and is based on person-centred ethics.
- The randomised controlled trial will be evaluated for effectiveness, health economic outcomes and process evaluation.
- Data will be analysed using quantitative and qualitative methods.
- The intervention has a generic design which makes it applicable also to other conditions, and to various categories of healthcare professionals.
- Because the study is mainly conducted in a research centre, future adaptions may be needed before implementing the intervention in other contexts and settings.

For peer review only - http://bmjopen.bmj.com/site/about/guidelines.xhtml

Introduction

As a major determinant of short- and long-term sick leave in many countries, mental illness bears a substantial portion of health care expenses and challenges (1-3). In Sweden, the total cost of mental illness has been estimated at 7.7 billion euros annually, of which about 50% is attributable to direct medical costs and 50% to work absence and out-of-work benefits (1). The number of sick leaves with origins from mental illness has increased during the past decade, and it is now the most common cause of sick leave in Sweden (4, 5). The conditions most frequently causing sick leave are depression, anxiety and stress-related disorders, which are generally referred to as common mental disorders (CMDs) (2, 6, 7). Sick leave attributable to CMDs has a long mean duration, with high risk of recurrent sickness episodes (7, 8). In Sweden, the majority of consultations concerning CMDs take place in primary care, and most patients are treated within primary care (1, 9). First-line treatment often consists of cognitive behavioural therapy, medication, or both (10), but the relationship between sickness duration and return to work (RTW) is complex (11-14). The existing evidence on interventions affecting RTW is limited; however, interventions that include a workplace component are more successful in improving RTW (15-19). In addition, interventions with multiple components appear more successful than single-component interventions (19). A recent systematic review by Etuknwa et al (20) evaluated which personal and social factors impacted RTW and found that self-efficacy, a positive attitude and support from leaders and co-workers were most relevant. The possible influence of self-efficacy on RTW has also been supported elsewhere (21, 22). Self-efficacy as a concept is rooted in social cognitive theory, which draws conclusions on human agency and its internal and external determinants (23). Perceived self-efficacy is a personal judgment of an individual's

For peer review only

capacity to handle general or specific situations in life, and as such, it is a core function of human agency. It is shaped by different sources of information and contextual factors; however, the best way to influence a person's self-efficacy is through successful experiences of mastery (24). Moreover, a strong sense of self-efficacy can reduce vulnerability to stressors and increase resilience to cope with adverse events (23, 25).

Self-efficacy is a central notion in person-centred care (PCC) (26-29). PCC can be conceptualised as initiatives advocating that healthcare systems need to treat and understand patients as persons (30). The concept of person is of vital importance in PCC as it transcends the more or less temporary and contextual role of being a patient. Persons have many features, characteristics and abilities in common, such as the ability to communicate and self-reflect, and at the same time, each person is someone in his or her own right with particular personal and circumstantial bodies, characteristics, abilities and histories (31, 32). For health care professionals (HCPs) to know their patients as persons, they need to be receptive to the unique premises of their patients' life. This approachability includes being sensitive to what is important to the patients, and neither diminish them on account of their vulnerability nor abandon them on account of their capabilities (30). To support HCPs and organisations to enhance person-centredness in clinical practice the Gothenburg Centre for Person-Centred Care (GPCC) proposed a framework to operationalise the ethics of PCC (29). In the framework a key factor in conducting PCC is the quality of communication and relationship, referred to as a partnership that includes evidence-based diagnostic procedures. Such a partnership is characterized by mutuality, respect, sharing and understanding, and is, at the same time, achieved through these means. Hence, a partnership is both the goal and a means to achieve PCC.

For peer review only - http://bmjopen.bmj.com/site/about/guidelines.xhtml

Previous evaluations of the GPCC framework in various conditions and care contexts have shown positive effects on several outcomes, including health-related quality of life (HRQoL) (33), patients' care experiences (34), uncertainty in illness (35), care costs (36, 37) and self-efficacy (26, 27). However, this approach has not yet been evaluated for CMDs. Combining PCC with an eHealth tool was shown to enhance further the ability to improve self-efficacy (38), and positive effects on self-efficacy have been demonstrated using telephone support (28). In addition, it appears that physical encounters are not essential to develop a patient-professional partnership (28, 39). Telehealth and eHealth solutions challenge the traditional structures in health care and hold possibilities of increasing transparency and accessibility. eHealth services may increase the possibility of patient involvement (e.g., empowerment and shared-decision making (40)), and psychological or behavioural eHealth interventions may, for example, contribute to a greater reach of patients who otherwise might not be subject to such assistance (41). This paper presents the design of a study evaluating a person-centred eHealth intervention for patients on sick leave due to CMDs. The primary aim is to evaluate the effects of a person-centred eHealth intervention (digital platform and telephone support) for patients on sick leave due to CMDs. Secondary aims are to evaluate the cost-effectiveness and the process of implementation to assess the fidelity of the eHealth intervention.

For peer review only

Methods and analysis

Study Design

The study is an open randomised controlled trial (RCT) with 1:1 allocation to either a control (standard care) or an intervention group (standard care plus person-centred eHealth intervention).

For peer review only - http://bmjopen.bmj.com/site/about/guidelines.xhtml

The trial was registered in the ClinicalTrials.gov (Identifier NCT03404583). Process evaluation and health economic analysis will also be conducted. The protocol is based on the SPIRIT (42) guidelines and the results will be reported according to established recommendations (43-45).

Study context

The study takes place in a larger city area in Western Sweden. Nine public primary health care centres are participating, all of which are located in a socioeconomically diverse area. The social insurance system in Sweden allows employees to take a maximum sick leave of 7 days without a medical certificate, and except for an initial day of "quarantine", they are financially covered by their employer during the first 14 days of illness. From day 8, a medical certificate is required that is usually obtained from a primary care or occupational health care physician. After 14 days of sick leave, benefits can be granted from the governmental Social Insurance Agency, which bases their decision upon the medical certificate.

Participants and recruitment

Recruitment of participants started in the spring of 2018 and is estimated to be completed during the fall of 2020. Patients on sick leave are consecutively screened in medical records by designated HCPs. Patients are eligible if they are on sick leave for no longer than 30 days due to a CMD that has been diagnosed by a physician. Table 1 presents the full inclusion and exclusion criteria. In the first step eligible patients receive a letter with brief information about the study. Next, they are invited to make contact for more information about the study or they will be called within the upcoming week for further information. Patients willing to participate will be sent more detailed information about the study and a consent form by mail. After written consent has been received, the patient is randomised to either the control or intervention group and informed

For peer review only - http://bmjopen.bmj.com/site/about/guidelines.xhtml

of the allocation. The randomisation is based on computer-generated random numbers and stratified by age (<50 or ≥50 years) and diagnostic group (depression, anxiety or stress reactions and disorders).

Table 1 Inclusion and exclusion criteria

Inclusion criteria	Exclusion criteria
• **Patients aged 18-65 years**<br>• **Currently employed or studying at least part-time during the past 9 months**<br>• Understand written and spoken Swedish<br>• Has a registered address<br>• **Currently on sick leave that has not exceeded 30 days, primarily due to any of the following diagnoses in ICD-10 as diagnosed by a physician: mild to moderate depression (F32 and F33), mild to moderate anxiety disorder (F41), reaction to severe stress and adjustment disorders (F43, except post-traumatic stress disorder. F43.8A Exhaustion disorder, a diagnosis in the Swedish ICD-10, is included).**	• Sick leave >14 days due to depression, anxiety disorders or stress reactions and disorders during the past 3 months<br>• Severe impairments hindering use of telephone or the digital platform<br>• Ongoing alcohol or drug abuse<br>• Any severe disease with an expected survival of <12 months or that can interfere with follow-up, or if the intervention is assessed as a burden<br>• Participating in a conflicting study

Patient and public involvement

For peer review only - http://bmjopen.bmj.com/site/about/guidelines.xhtml

Public involvement is an essential part of the research conducted at GPCC. The development of the digital platform was guided by a participatory design (46). An advisory group comprising potential end-users (e.g., patient-representatives and HCPs) offered suggestions to the design of the digital platform. These end-users will be continuously consulted throughout the study period (e.g., in developing an interview guide and discussing dissemination).

Control group

Standard care commonly involves meetings with a physician to follow up on decisions regarding sick leave and RTW, as well as advice and information on self-care, but may encompass medication or cognitive behavioural therapy (10). Referral to other health care professions depends upon individual assessment and can include meetings with a physiotherapist, psychologist or psychotherapist, occupational therapist, group treatments, etc. Involving the workplace in an early, but timely stage is important regarding RTW.

Intervention group

In addition to standard care, patients allocated to the intervention group will receive PCC via telephone and a digital platform. The structure of the intervention is described in table 2. Following allocation, patients will be e-mailed a link to create an account and access the digital platform. They will be contacted by an HCP to schedule a time for an initial PCC conversation by telephone. This conversation, which is preferably scheduled within 1 week after initial contact, aspires to create a jointly agreed health plan, one that will serve as a foundation for future collaboration during the intervention period. A team of health care personnel with different professional backgrounds (e.g., registered nurses and physiotherapists) are involved in conducting the intervention. All received a half-day introduction to CMDs and the philosophical

For peer review only - http://bmjopen.bmj.com/site/about/guidelines.xhtml

underpinnings of PCC, and training in how to apply PCC in practice, e.g., through active listening, asking open-ended questions, jointly reflecting and providing summaries. They also have a forum to meet regularly with each other and specialists in the area throughout the intervention in which they can raise questions about the intervention as well as the practice of conducting care based on person-centred ethics. In this forum the HCPs will also share and discuss some of the health plans and telephone calls they have conducted, as part of their training and continuous development of skills in person-centred communication.

Table 2 Content of intervention components

Telephone	Digital platform
• **HCP's initiate a first telephone conversation shortly after inclusion**	On the platform, patients can:<br>• Write and read health plans<br>• Communicate with HCP in a chat-like forum<br>• Seek information on their condition<br>• Invite significant others, such as family members and workplace representatives, to take part of their platform content<br>• Make daily notes and ratings on symptoms and general well-being, and visualize answers in the form of trend graphs
• **Following telephone communication is scheduled according to agreement but patients can also contact HCPs spontaneously**	• Patients can decide which functions of the platform they want the invited persons to access, and can both add new and delete persons at all times
• **The content of the telephone conversation(s) is central to what is documented in the patient's health plan, which is uploaded to the platform**	• If needed, HCPs will guide patients on how to access the platform. Patients are encouraged but not obliged to use the platform

HCP = Health Care Professionals

For peer review only - http://bmjopen.bmj.com/site/about/guidelines.xhtml

Telephone support

In the telephone conversations the patient narrative is central and the HCP will be attentive to the expressions of needs, goals, resources and experiences of the patients' current situation as well as their health status (26, 28). There is no fixed manual for how these conversations should develop nor any topics which they must cover. However, in each conversation, HCPs are asking questions and listening to the patient with the intent to understand the patient's experiences of their condition in the context of their everyday life. Together with the patient they discuss what could be achievable and desirable goals for the near future. They collaborate with the patient to identify their strengths and resources, for example by inquiring on how the patient has previously handled challenging situations. The patient's narrative is then documented in a health plan by either the patient or the HCP, and uploaded to the digital platform. The health plan should capture the patients' experiences of their situation and what health-related changes they want to achieve and how, including what resources they have in terms of personal capabilities and surrounding support systems. This health plan will serve as a guide in future contacts between the HCPs and the patient. It can be modified or reformulated according to what occurs during the intervention and the patient's process. Hence, the health plan is a living document negotiated in collaboration and documented such that both the HCPs and patients (and preferably the extended network of the patients' choice) have continuous access. This is what all patients participating in the intervention will receive, at a minimum, and this procedure builds upon the GPCC framework to PCC (29). How the support will be conducted after this first telephone call is discussed and planned in a mutual agreement between HCPs and patients. Different HCPs can interact with the same patient during the intervention, depending on scheduling matters. If that is the case, the documentation of the health plan enables a continuity, so the patients don't have to restart their

For peer review only - http://bmjopen.bmj.com/site/about/guidelines.xhtml

narrative process. However, if patients explicitly wish to continue interacting with the same HCP this is arranged for, if possible.

The digital platform

The digital platform was constructed to be non-directive and create possibilities for patients to take an active part in their recovery and rehabilitation, which is an important objective in PCC. Initially, patients and HCPs have access to the platform. Patients can also choose to expand their network by inviting people of importance to them or their rehabilitation, such as family members, other health care contacts or workplace representatives. The platform should function as a mediator through which the patients, together with their network, can monitor symptoms and be informed of their recovery process or risk of relapse or deterioration. The platform provides patients with the possibility to generate daily notes on symptoms and general wellbeing. By rating on a scale from 1-5, e.g. how well they have slept or how well they perceive their ability to concentrate, their answers will be visualised as graphs, which will provide the possibility to see trends and development over time. The patients can take private notes about their ratings and hence this may function in a diary-like manner. In addition, the patients and the HCPs can communicate with each other through messages in a chat-like forum and the platform contains an assembly of links to other web pages on CMD that the patients can use to seek information or connections. HCPs log in to the platform at least once a day to be updated on patients' activities and check for messages. When patients invite someone to their platform page, they can decide which functions of the platform they want the invited person to access. Patients can both add new persons or delete persons that have access to the account. The platform can be accessed from any device with an internet connection and web browser, such as a computer, smartphone or iPad.

For peer review only - http://bmjopen.bmj.com/site/about/guidelines.xhtml

Outcomes and measurements

Questionnaire data are gathered at baseline and after 3, 6, 12, 18 and 24 months from the inclusion date. Self-reported baseline characteristics of the participants' sex, age, civil status, country of birth, level of education, occupation and number of working years are collected through questionnaires sent by mail. Additional baseline characteristics (e.g., income) will be gathered from the Longitudinal Integration Database for Health Insurance and Labour Market Studies (LISA).

Primary outcome

The primary outcome measure is a composite score of changes in sick leave and general self-efficacy (26, 28, 47). Patients will be classified as improved, deteriorated or unchanged according to the following standards: if the patient has a reduced sick leave percentage and an increased general self-efficacy by ≥5 units at the 6-month follow-up, the patient is classified as improved. If the patient has an increased percentage of sick leave absence or a reduced general self-efficacy by ≥5 units at the 6-month follow-up, the patient is classified as deteriorated. Those who are neither deteriorated nor improved are considered unchanged. Sick leave will be regarded from two perspectives: the duration of the initial absence and the total absence after 24 months from the inclusion date. Data on full- and part-time sick leave (25/50/75% of full-time) will be both self-reported by the participants through questionnaires and gathered from the Micro Data for the Analysis of Social Insurance register (MiDAS). Self-efficacy will be measured using the General Self-Efficacy Scale (GSE) (48, 49), a 10-item self-assessment questionnaire measuring a person's general sense of competence towards dealing with unforeseen situations. Responses are made on a four-point scale (1 = not at all true, 2 = hardly true, 3 = moderately true, 4 = exactly true), with

For peer review only - http://bmjopen.bmj.com/site/about/guidelines.xhtml

a composite score based on the sum of all items. The total score ranges from 10 to 40, with higher scores indicating a higher sense of self-efficacy.

Secondary outcomes

Additional analyses will be conducted and based on data from the following instruments:

- General self-efficacy (48, 49)
- EuroQol 5-Dimension health state questionnaire (EQ-5D) (50)
- Shirom-Melamed Burnout Questionnaire (SMBQ) (51)
- Perceived Stress Scale (PSS) (52)
- Self-rated Exhaustion Disorder (s-ED) (53)
- Hospital Anxiety and Depression Scale (HADS) (54)
- Multidimensional Fatigue Symptom Inventory (MFI-20) (55)
- Self-Efficacy for Managing Chronic Disease 6-item Scale (SEMCD) (56)
- Sheehan Disability Scale (SDS) (57)
- Health care utilization
- Incremental cost-utility ratios
- Sick leave

Power/Sample size

To achieve a power of 80% based on a one-sided alpha error of 0.05 91 participants would be needed in each study group to detect an improvement in the composite score (20% in the control

For peer review only

For peer review only - http://bmjopen.bmj.com/site/about/guidelines.xhtml

group vs. 40% in the intervention group). We aim to include a minimum of 110 patients per group to have some margin for dropouts or withdrawals.

Data Analysis

Descriptive and analytic statistics will be conducted to compare the study groups. Logistic regression will be used on the primary outcome measure to calculate odds ratios with 95% confidence intervals (CIs). For highly skewed data (e.g., cost data), a bootstrap method will be used to estimate CIs (58). Parametric and nonparametric statistics will be carried out for secondary outcome measures. Intention-to-treat and, if needed, per-protocol analyses will be performed. Subgroup analyses will be conducted on, e.g., sex, age and diagnostic groups.

Economic evaluation

The economic evaluation will assess whether the cost of introducing this add-on PCC intervention is acceptable compared with the achieved health effects e.g., measured as HRQoL collected through questionnaires. Missing questionnaire data will be assumed missing-at-random and thus handled using multiple imputations. The health state index derived from the EQ-5D questionnaire will be translated into quality-adjusted life-years using an area under the curve calculation and the Swedish experience-based valuation (59) and, as a sensitivity analysis, the society-based valuation for the UK (60). The calculation of costs from a societal perspective will include costs for prescribed drugs, health care encounters and lost productivity. Drug costs will be obtained from the Swedish Prescribed Drug Register, held by the National Board of Health and Welfare. Health care encounters will be obtained from the regional patient register VEGA and converted to costs using diagnosis-related group (DRG) weights (available for all encounters in specialised health care) and the national cost per one DRG, whereas unit costs presented in

For peer review only - http://bmjopen.bmj.com/site/about/guidelines.xhtml

national statistics will be used for primary care contacts (since DRGs are not available for primary care). In addition, we will collect information about the time spent by HCPs interacting with participants on the platform and by phone. Lost productivity will be obtained from both questionnaires and the MiDAS register, under the authority of the Social Insurance Agency, to ensure all absenteeism is recorded. This is done because the national register only fully covers days off from work that are reimbursed by the agency. Indirect costs for lost productivity will then be calculated using "the human capital approach", which includes multiplying time off from work by mean wages (by age groups) and the social security contribution paid by employers (61). Total costs and costs per payer category (patients, market sector, health care providers/regions and other authorities/tax-based funds) will be reported. The incremental cost will then be compared with incremental health effects using the incremental cost-effectiveness ratio (ICER). To explore the value of the intervention the ICER will be compared with the informal cost-effectiveness threshold used in Sweden of SEK 500,000 and to alternative willingness-to-pay thresholds using the cost-effectiveness acceptability curve.

Process evaluation

To link the outcomes of the intervention to the implementation process we will explore three complementary aspects of the intervention: 1) how much and in what way participants have used the PCC intervention, 2) how they experienced it and 3) whether it has been used as intended (62). Quantitative data will be collected from survey questions on the use and ratings of telephone and platform support, which will be attached to the 3- and 6-month questionnaires sent to those in the intervention group. We will also collect data on the use of different functions of the intervention and number and modes of contact between HCPs, patients and persons invited to the platform during the intervention period. Qualitative data will consist of transcribed audio

For peer review only - http://bmjopen.bmj.com/site/about/guidelines.xhtml

recordings of telephone conversations between HCPs and patients. In addition, open questions in the 3-and 6-month surveys permit elaboration on experiences of the activities and delivery of the intervention. We will also interview a sample of patients in the intervention group, as well as involved HCPs and persons invited to the platform for an in-depth exploration of their experiences of the intervention. Qualitative data will be analysed using qualitative analysis methods and quantitative data using descriptive statistics. Using both quantitative and qualitative data allows applying mixed-methods analysis to triangulate the data, which increases the reliability of the study outcome.

Ethics and dissemination

The study was approved by the Regional Ethical Review Board in Gothenburg, Sweden (DNr 497-17, T 023-18, T 526-18). All participants agreed to participate and signed a written consent form after receiving written and oral information about the study. Informed consent will also be asked of participants accepting to be interviewed. Studies in the project will be published in peer-reviewed scientific journals and presented at national and international conferences. This project is part of a research programme at the GPCC, where extensive work is ongoing to disseminate knowledge on and implementation of PCC.

Discussion

The person-centred health care process is based in a continuous negotiation to understand the general and unique premises of each patient's situation, with decisions and actions guided by that understanding (30, 63). In the present study, this is operationalized in the communication between HCPs and patients. The objective of the telephone conversations is for the patients to

For peer review only

communicate their perspective regarding their condition and its effect on everyday life. The HCP engage in the patient's narrative by active listening and asking open-ended question, and together they set goals derived from this communication (29). Consequently, PCC and person-centred interventions are complex, as they are individually tailored rather than following a standardised procedure. In the present study, the participants will use the different features of the intervention according to their individual needs. How the intervention has been conducted will be monitored and clarified in the process evaluation, which is an essential exploration in order to understand the pathways of a complex intervention (62). Hawe et al (64) suggest that process evaluations of complex interventions needs to clearly state what is assumed to be the essential element of change. As long as the essential elements are not altered, other intervention components can be adapted to serve the local context. Applied to the present study, we assume that the essential element to generate change is the quality of the person-centred communication. Considering the population of primary care patients on sick leave due to a CMD, part of the recent increase in mental illness-related sick leaves is possibly explained by both work-related and private life stress exposure (65). Psychosocial stress exposure can be conceptualised as an imbalance between demands, support and resources such as control, including both work and private life situation (66). Consequently, we believe that it is essential with a person-centred communication rooted in an understanding of the patient as both vulnerable and capable and that reaching agreements on care and rehabilitation build upon the knowledge of the patient's circumstances, needs, wishes and resources. We included self-efficacy as a primary outcome measure for the RCT. This choice is based mainly on two reasons. First, several studies have suggested self-efficacy as an important psychological resource in an RTW process (20-22). Second, studies on PCC interventions have reported positive effects of self-efficacy (26-28, 38).

For peer review only - http://bmjopen.bmj.com/site/about/guidelines.xhtml

The study design has some limitations. Although the study is conducted in cooperation with primary care centres, the intervention is mainly performed by designated HCPs situated in a research centre in a separate location. One advantage of that arrangement is the possibility to follow the process closely thanks to full access to all patient-staff communication. However, the study results will have to be considered in light of that and future adaptions may be needed before implementing the intervention in other contexts and settings. Another limitation is the language restriction. It has not been feasible to involve interpreters and therefore only patients who can independently manage to communicate in Swedish will be included in the study. Participation also require access to an internet device. Furthermore, although we consider it important from a perspective of PCC not to impose a certain standardised procedure of the intervention upon participants, it is also possible that participants will not use the intervention to its full extent, which we consider a limitation. For example, although earlier research show the importance of workplace involvement to impact on RTW (15-19), this is included in the PROMISE intervention as a voluntary feature. Although the patients should be encouraged to invite their network to the platform, it is possible that the voluntary design will result in them doing so to a lesser degree than desired. Hopefully, the intervention components perceived as helpful by the participants, will also be applied to a desired extent (67). It is also possible that the designation of a 5-point difference in general self-efficacy, which has proved relevant in other clinical settings (26, 28), will not be valid for common mental disorders.

The present study corresponds to the current call for interventions targeting sick leave in CMD patients. The design of the intervention is consistent with what previous reviews have shown promising, e.g., facilitating collaboration, using multiple components (19) and providing possibilities of involving the workplace (15-19). The choice to develop and evaluate an eHealth

For peer review only - http://bmjopen.bmj.com/site/about/guidelines.xhtml

intervention was largely because of the ambition to limit the number of physical appointments the patients would need to attend and reach the patients in their everyday life at home. We believe this represents one of the potential benefits of eHealth services. However, it is fundamental that also remotely delivered health care is person-centred. We also believe that developing interventions, which are not limited to a certain professional category and targeting not only one diagnosis, is necessary considering the current pressure on first-line health care facilities for CMDs. We consider it a strength that our study investigates PCC as an addition to, not in comparison with, standard care. This intervention approach is expected to gain new knowledge on how PCC can contribute to the challenges facing healthcare systems with a large number of persons seeking help for their mental illness and the difficulties in coping with work and everyday life.

References

1. OECD. Mental Health and Work: Sweden. Paris: OECD Publishing; 2013.
2. Henderson M, Glozier N, Elliott KH. Long term sickness absence. BMJ. 2005;330(7495):802-3.
3. Henderson M, Harvey SB, Øverland S, Mykletun A, Hotopf M. Work and common psychiatric disorders. London, England2011. p. 198-207.
4. Försäkringskassan [Swedish Social Insurance Agency]. Social Insurance in Figures 2019. https://www.forsakringskassan.se/wps/wcm/connect/cec4cea8-1d6c-4895-b442-bc3b64735b09/socialforsakringen-i-siffror-2019-engelsk.pdf?MOD=AJPERES&CVID=2019.
5. Försäkringskassan [Swedish Social Insurance Agency]. Uppföljning av sjukfrånvarons utveckling 2019. https://wwwforsakringskassanse/wps/wcm/connect/fb32bcf5-7ed4-4356-b18b-6adc7ca20fcd/1573-19-svar-regeringsuppdragpdf?MOD=AJPERES&CVID=. https://www.forsakringskassan.se/wps/wcm/connect/fb32bcf5-7ed4-4356-b18b-6adc7ca20fcd/1573-19-svar-regeringsuppdrag.pdf?MOD=AJPERES&CVID=2019.
6. Lidwall U. Sick leave diagnoses and return to work: a Swedish register study. Disability and Rehabilitation. 2015;37(5):396-410.
7. Försäkringskassan [Swedish Social Insurance Agency]. Sjukfrånvarons utveckling 2017. https://www.forsakringskassan.se/wps/wcm/connect/1596d32b-7ff7-4811-8215-d90cb9c2f38d/socialforsakringsrapport-2017-13.pdf?MOD=AJPERES&CVID=2017.
8. Dewa CS, Loong D, Bonato S. Work outcomes of sickness absence related to mental disorders: A systematic literature review. BMJ Open. 2014;4(7).

For peer review only - http://bmjopen.bmj.com/site/about/guidelines.xhtml

9. Sundquist J, Ohlsson H, Sundquist K, Kendler KS. Common adult psychiatric disorders in Swedish primary care where most mental health patients are treated. BMC Psychiatry. 2017;17(1):235.
10. Socialstyrelsen [National Board of Health and Welfare]. Nationella riktlinjer för vård vid depression och ångestsyndrom. Socialstyrelsen; 2017.
11. Soegaard H. Variation in effect of intervention studies in research on sickness absence. Open Access Journal of Clinical Trials. 2012;4:1-20.
12. Nieuwenhuijsen K, Bültmann U, Neumeyer-Gromen A, Verhoeven AC, Verbeek JH, Feltz-Cornelis CM. Interventions to improve occupational health in depressed people. Cochrane Database of Systematic Reviews. 2008(2).
13. van der Klink JJL, Blonk RWB, Schene AH, van Dijk FJH. Reducing long term sickness absence by an activating intervention in adjustment disorders: a cluster randomised controlled design. Occupational and Environmental Medicine. 2003;60(6):429.
14. Blank L, Peters J, Pickvance S, Wilford J, MacDonald E. A Systematic Review of the Factors which Predict Return to Work for People Suffering Episodes of Poor Mental Health. Journal of Occupational Rehabilitation. 2008;18(1):27-34.
15. Vogel N, Schandelmaier S, Zumbrunn T, Ebrahim S, de Boer WE, Busse JW, et al. Return-to-work coordination programmes for improving return to work in workers on sick leave. Cochrane Database Syst Rev. 2017;3:Cd011618.
16. van Vilsteren M, van Oostrom SH, de Vet HC, Franche RL, Boot CR, Anema JR. Workplace interventions to prevent work disability in workers on sick leave. Cochrane Database Syst Rev. 2015(10):Cd006955.
17. Arends I, Bruinvels DJ, Rebergen DS, Nieuwenhuijsen K, Madan I, Neumeyer-Gromen A, et al. Interventions to facilitate return to work in adults with adjustment disorders. Cochrane Database Syst Rev. 2012;12:Cd006389.
18. Nieuwenhuijsen K, Faber B, Verbeek JH, Neumeyer-Gromen A, Hees HL, Verhoeven AC, et al. Interventions to improve return to work in depressed people. Cochrane Database Syst Rev. 2014(12):Cd006237.
19. Mikkelsen MB, Rosholm M. Systematic review and meta-analysis of interventions aimed at enhancing return to work for sick-listed workers with common mental disorders, stress-related disorders, somatoform disorders and personality disorders. Occup Environ Med. 2018;75(9):675-86.
20. Etuknwa A, Daniels K, Eib C. Sustainable Return to Work: A Systematic Review Focusing on Personal and Social Factors. Journal of Occupational Rehabilitation. 2019.
21. Volker D, Zijlstra-Vlasveld MC, Brouwers EP, van Lomwel AG, van der Feltz-Cornelis CM. Return-to-Work Self-Efficacy and Actual Return to Work Among Long-Term Sick-Listed Employees. J Occup Rehabil. 2015;25(2):423-31.
22. Nigatu Y, Liu Y, Uppal M, McKinney S, Gillis K, Rao S, et al. Prognostic factors for return to work of employees with common mental disorders: a meta-analysis of cohort studies. Social Psychiatry and Psychiatric Epidemiology. 2017;52(10):1205-15.
23. Bandura A. Social cognitive theory: an agentic perspective. Annu Rev Psychol. 2001;52:1-26.
24. Bandura A. Self-efficacy: toward a unifying theory of behavioral change. Psychol Rev. 1977;84(2):191-215.
25. Seggelen - Damen Iv, Dam Kv. Self-reflection as a mediator between self-efficacy and well-being. Journal of Managerial Psychology. 2016;31(1):18-33.

For peer review only - http://bmjopen.bmj.com/site/about/guidelines.xhtml

26. Fors A, Ekman I, Taft C, Bjorkelund C, Frid K, Larsson ME, et al. Person-centred care after acute coronary syndrome, from hospital to primary care - A randomised controlled trial. Int J Cardiol. 2015;187:693-9.
27. Fors A, Taft C, Ulin K, Ekman I. Person-centred care improves self-efficacy to control symptoms after acute coronary syndrome: a randomized controlled trial. European Journal of Cardiovascular Nursing. 2016;15(2):186-94.
28. Fors A, Blanck E, Ali L, Ekberg-Jansson A, Fu M, Kjellberg IL, et al. Effects of a person-centred telephone-support in patients with chronic obstructive pulmonary disease and/or chronic heart failure - A randomized controlled trial. Plos One. 2018;13(8).
29. Ekman I, Swedberg K, Taft C, Lindseth A, Norberg A, Brink E, et al. Person-centered care--ready for prime time. Eur J Cardiovasc Nurs. 2011;10(4):248-51.
30. Entwistle VA, Watt IS. Treating Patients as Persons: A Capabilities Approach to Support Delivery of Person-Centered Care. The American Journal of Bioethics. 2013;13(8):29-39.
31. Leplege A, Gzil F, Cammelli M, Lefeve C, Pachoud B, Ville I. Person-centredness: conceptual and historical perspectives. Disability and rehabilitation. 2007;29(20-21):1555.
32. Mccormack B, Mccance, T Personhood and Person-Centredness. Person-Centred Nursing. Oxford, UK: Wiley-Blackwell; 2010. p. 5-20.
33. Brannstrom M, Boman K. Effects of person-centred and integrated chronic heart failure and palliative home care. PREFER: a randomized controlled study. Eur J Heart Fail. 2014;16(10):1142-51.
34. Wolf A, Vella R, Fors A. The impact of person-centred care on patients' care experiences in relation to educational level after acute coronary syndrome: secondary outcome analysis of a randomised controlled trial. Eur J Cardiovasc Nurs. 2019;18(4):299-308.
35. Dudas K, Olsson LE, Wolf A, Swedberg K, Taft C, Schaufelberger M, et al. Uncertainty in illness among patients with chronic heart failure is less in person-centred care than in usual care. Eur J Cardiovasc Nurs. 2013;12(6):521-8.
36. Hansson E, Ekman I, Swedberg K, Wolf A, Dudas K, Ehlers L, et al. Person-centred care for patients with chronic heart failure - a cost-utility analysis. Eur J Cardiovasc Nurs. 2016;15(4):276-84.
37. Gyllensten H, Koinberg I, Carlstrom E, Olsson LE, Hansson Olofsson E. Economic evaluation of a person-centred care intervention in head and neck oncology: results from a randomized controlled trial. Support Care Cancer. 2019;27(5):1825-34.
38. Wolf A, Fors A, Ulin K, Thorn J, Swedberg K, Ekman I. An eHealth Diary and Symptom-Tracking Tool Combined With Person-Centered Care for Improving Self-Efficacy After a Diagnosis of Acute Coronary Syndrome: A Substudy of a Randomized Controlled Trial. J Med Internet Res. 2016;18(2):e40.
39. Heckemann B, Wolf A, Ali L, Sonntag SM, Ekman I. Discovering untapped relationship potential with patients in telehealth: a qualitative interview study. BMJ Open. 2016;6(3):e009750.
40. Eysenbach G. What is e-health? J Med Internet Res. 2001;3(2):e20.
41. Andersson G. Internet-Delivered Psychological Treatments. Annual Review of Clinical Psychology. 2016;12(1):157-79.
42. Chan A-W, Tetzlaff JM, Gøtzsche PC, Altman DG, Mann H, Berlin JA, et al. SPIRIT 2013 explanation and elaboration: guidance for protocols of clinical trials. BMJ : British Medical Journal. 2013;346:e7586.

For peer review only - http://bmjopen.bmj.com/site/about/guidelines.xhtml

43. Moher D, Schulz KF, Altman DG. The CONSORT statement: revised recommendations for improving the quality of reports of parallel-group randomised trials. Lancet. 2001;357(9263):1191-4.
44. Husereau D, Drummond M, Petrou S, Carswell C, Moher D, Greenberg D, et al. Consolidated Health Economic Evaluation Reporting Standards (CHEERS) statement. BMJ : British Medical Journal. 2013;346:f1049.
45. Pinnock H, Barwick M, Carpenter CR, Eldridge S, Grandes G, Griffiths CJ, et al. Standards for Reporting Implementation Studies (StaRI): explanation and elaboration document. BMJ Open. 2017;7(4):e013318.
46. Sanders EBN, Stappers PJ. Co-creation and the new landscapes of design. CoDesign. 2008;4(1):5-18.
47. Packer M. Proposal for a new clinical end point to evaluate the efficacy of drugs and devices in the treatment of chronic heart failure. J Card Fail. 2001;7(2):176-82.
48. Schwarzer R, & Jerusalem, M. . Generalized Self-Efficacy scale. In: Johnston M, Wright, S., Weinman, J. , editor. Measures in health psychology: A user's portfolio. Windsor: UK: NFER-NELSON; 1995. p. 35-7.
49. Love J, Moore CD, Hensing G. Validation of the Swedish translation of the General Self-Efficacy scale. Qual Life Res. 2012;21(7):1249-53.
50. Brooks R. EuroQol: the current state of play. Health policy. 1996;37(1):53-72.
51. Lundgren-Nilsson A, Jonsdottir IH, Pallant J, Ahlborg G, Jr. Internal construct validity of the Shirom-Melamed Burnout Questionnaire (SMBQ). BMC Public Health. 2012;12:1.
52. Cohen S, Kamarck T, Mermelstein R. A global measure of perceived stress. J Health Soc Behav. 1983;24(4):385-96.
53. Glise K, Hadzibajramovic E, Jonsdottir IH, Ahlborg G, Jr. Self-reported exhaustion: a possible indicator of reduced work ability and increased risk of sickness absence among human service workers. Int Arch Occup Environ Health. 2010;83(5):511-20.
54. Zigmond AS, Snaith RP. The hospital anxiety and depression scale. Acta Psychiatr Scand. 1983;67(6):361-70.
55. Smets EM, Garssen B, Bonke B, De Haes JC. The Multidimensional Fatigue Inventory (MFI) psychometric qualities of an instrument to assess fatigue. J Psychosom Res. 1995;39(3):315-25.
56. Lorig KR, Sobel DS, Ritter PL, Laurent D, Hobbs M. Effect of a self-management program on patients with chronic disease. Eff Clin Pract. 2001;4(6):256-62.
57. Leon AC, Olfson M, Portera L, Farber L, Sheehan DV. Assessing psychiatric impairment in primary care with the Sheehan Disability Scale. Int J Psychiatry Med. 1997;27(2):93-105.
58. Barber JA, Thompson SG. Analysis of cost data in randomized trials: an application of the non-parametric bootstrap. Statistics in Medicine. 2000;19(23):3219-36.
59. Burström K, Sun S, Gerdtham U-G, Henriksson M, Johannesson M, Levin L-Å, et al. Swedish experience-based value sets for EQ-5D health states. Quality of Life Research. 2014;23(2):431-42.
60. Dolan P, Gudex C, Kind P, Williams A. A social tariff for EuroQol: results from a UK general population survey. 1995.
61. Krol M, Brouwer W, Rutten F. Productivity Costs in Economic Evaluations: Past, Present, Future. PharmacoEconomics. 2013;31(7):537-49.

For peer review only

For peer review only - http://bmjopen.bmj.com/site/about/guidelines.xhtml

62. Moore GF, Audrey S, Barker M, Bond L, Bonell C, Hardeman W, et al. Process evaluation of complex interventions: Medical Research Council guidance. BMJ : British Medical Journal. 2015;350:h1258.
63. McCormack B, McCance T. Person-centred practice in nursing and health care : theory and practice. Second edition ed: Chichester : Wiley Blackwell; 2017.
64. Hawe P, Shiell A, Riley T. Complex interventions: how "out of control" can a randomised controlled trial be? BMJ. 2004;328(7455):1561-3.
65. Hasselberg K, Jonsdottir IH, Ellbin S, Skagert K. Self-reported stressors among patients with exhaustion disorder: an exploratory study of patient records. BMC psychiatry. 2014;14(1):66.
66. Karasek R. Healthy work : stress, productivity, and the reconstruction of working life. Theorell T, editor. New York, N.Y.: New York, N.Y. : Basic Books; 1990.
67. Beatty L, Binnion C. A Systematic Review of Predictors of, and Reasons for, Adherence to Online Psychological Interventions. International Journal of Behavioral Medicine. 2016;23(6):776-94.

Author Contributions

All authors were involved in the design of the study. MC drafted the manuscript with critical input from LA, IE, KG, IJ, HG, KS and AF. AF is the principal investigator and grant holder of the investigation. All authors reviewed, edited and approved the final version of the manuscript.

Funding statement

This work was supported by The Swedish Research Council for Health, Working Life and Welfare (reference number 2016-07418, 2017-00557 and 2019-01726). The funder has no role in the design of the study, data collection, analysis, or interpretation. The study was financed by grants from the Swedish state under the agreement between the Swedish government and the country councils, the ALF agreement (ALFGBG-772191 and ALFGBG-932659).

Competing interests' statement

Nothing to declare

For peer review only - http://bmjopen.bmj.com/site/about/guidelines.xhtml

Word count

4303

For peer review only - http://bmjopen.bmj.com/site/about/guidelines.xhtml

Reporting checklist for protocol of a clinical trial.

Based on the SPIRIT guidelines.

Instructions to authors

Complete this checklist by entering the page numbers from your manuscript where readers will find each of the items listed below.

Your article may not currently address all the items on the checklist. Please modify your text to include the missing information. If you are certain that an item does not apply, please write "n/a" and provide a short explanation.

Upload your completed checklist as an extra file when you submit to a journal.

In your methods section, say that you used the SPIRITreporting guidelines, and cite them as:

Chan A-W, Tetzlaff JM, Altman DG, Laupacis A, Gøtzsche PC, Krleža-Jerić K, Hróbjartsson A, Mann H, Dickersin K, Berlin J, Doré C, Parulekar W, Summerskill W, Groves T, Schulz K, Sox H, Rockhold FW, Rennie D, Moher D. SPIRIT 2013 Statement: Defining standard protocol items for clinical trials. Ann Intern Med. 2013;158(3):200-207

		Reporting Item	Page Number
**Administrative information**			
Title	#1	Descriptive title identifying the study design, population, interventions, and, if applicable, trial acronym	1
Trial registration	#2a	Trial identifier and registry name. If not yet registered, name of intended registry	6
Trial registration: data set	#2b	All items from the World Health Organization Trial Registration Data Set	n/a
Protocol version	#3	Date and version identifier	n/a
Funding	#4	Sources and types of financial, material, and other support	21-22
Roles and responsibilities: contributorship	#5a	Names, affiliations, and roles of protocol contributors	21

For peer review only

Roles and responsibilities: sponsor contact information	#5b	Name and contact information for the trial sponsor	n/a
Roles and responsibilities: sponsor and funder	#5c	Role of study sponsor and funders, if any, in study design; collection, management, analysis, and interpretation of data; writing of the report; and the decision to submit the report for publication, including whether they will have ultimate authority over any of these activities	21-22
Roles and responsibilities: committees	#5d	Composition, roles, and responsibilities of the coordinating centre, steering committee, endpoint adjudication committee, data management team, and other individuals or groups overseeing the trial, if applicable (see Item 21a for data monitoring committee)	n/a
**Introduction**			
Background and rationale	#6a	Description of research question and justification for undertaking the trial, including summary of relevant studies (published and unpublished) examining benefits and harms for each intervention	3-5
Background and rationale: choice of comparators	#6b	Explanation for choice of comparators	8
Objectives	#7	Specific objectives or hypotheses	5
Trial design	#8	Description of trial design including type of trial (eg, parallel group, crossover, factorial, single group), allocation ratio, and framework (eg, superiority, equivalence, non-inferiority, exploratory)	6
**Methods: Participants, interventions, and outcomes**			
Study setting	#9	Description of study settings (eg, community clinic, academic hospital) and list of countries where data will be collected. Reference to where list of study sites can be obtained	6
Eligibility criteria	#10	Inclusion and exclusion criteria for participants. If applicable, eligibility criteria for study centres and individuals who will	6-7

For peer review only

		perform the interventions (eg, surgeons, psychotherapists)	
Interventions: description	#11a	Interventions for each group with sufficient detail to allow replication, including how and when they will be administered	8-10
Interventions: modifications	#11b	Criteria for discontinuing or modifying allocated interventions for a given trial participant (eg, drug dose change in response to harms, participant request, or improving / worsening disease)	n/a
Interventions: adherance	#11c	Strategies to improve adherence to intervention protocols, and any procedures for monitoring adherence (eg, drug tablet return; laboratory tests)	n/a
Interventions: concomitant care	#11d	Relevant concomitant care and interventions that are permitted or prohibited during the trial	n/a
Outcomes	#12	Primary, secondary, and other outcomes, including the specific measurement variable (eg, systolic blood pressure), analysis metric (eg, change from baseline, final value, time to event), method of aggregation (eg, median, proportion), and time point for each outcome. Explanation of the clinical relevance of chosen efficacy and harm outcomes is strongly recommended	10-12
Participant timeline	#13	Time schedule of enrolment, interventions (including any run-ins and washouts), assessments, and visits for participants. A schematic diagram is highly recommended (see Figure)	6
Sample size	#14	Estimated number of participants needed to achieve study objectives and how it was determined, including clinical and statistical assumptions supporting any sample size calculations	12
Recruitment	#15	Strategies for achieving adequate participant enrolment to reach target sample size	n/a
**Methods: Assignment of interventions (for controlled trials)**			
Allocation: sequence generation	#16a	Method of generating the allocation sequence (eg, computer-generated random numbers), and list of any factors for stratification. To reduce predictability of a random sequence, details of any planned restriction (eg, blocking) should be provided in a separate document that is unavailable to those who enrol participants or assign interventions	7

For peer review only

Allocation concealment mechanism	#16b	Mechanism of implementing the allocation sequence (eg, central telephone; sequentially numbered, opaque, sealed envelopes), describing any steps to conceal the sequence until interventions are assigned	n/a
Allocation: implementation	#16c	Who will generate the allocation sequence, who will enrol participants, and who will assign participants to interventions	6
Blinding (masking)	#17a	Who will be blinded after assignment to interventions (eg, trial participants, care providers, outcome assessors, data analysts), and how	n/a
Blinding (masking): emergency unblinding	#17b	If blinded, circumstances under which unblinding is permissible, and procedure for revealing a participant's allocated intervention during the trial	n/a
**Methods: Data collection, management, and analysis**			
Data collection plan	#18a	Plans for assessment and collection of outcome, baseline, and other trial data, including any related processes to promote data quality (eg, duplicate measurements, training of assessors) and a description of study instruments (eg, questionnaires, laboratory tests) along with their reliability and validity, if known. Reference to where data collection forms can be found, if not in the protocol	10-14
Data collection plan: retention	#18b	Plans to promote participant retention and complete follow-up, including list of any outcome data to be collected for participants who discontinue or deviate from intervention protocols	n/a
Data management	#19	Plans for data entry, coding, security, and storage, including any related processes to promote data quality (eg, double data entry; range checks for data values). Reference to where details of data management procedures can be found, if not in the protocol	n/a
Statistics: outcomes	#20a	Statistical methods for analysing primary and secondary outcomes. Reference to where other details of the statistical analysis plan can be found, if not in the protocol	12-14
Statistics: additional analyses	#20b	Methods for any additional analyses (eg, subgroup and adjusted analyses)	12-14

For peer review only

Statistics: analysis population and missing data	#20c	Definition of analysis population relating to protocol non-adherence (eg, as randomised analysis), and any statistical methods to handle missing data (eg, multiple imputation)	13
**Methods: Monitoring**			
Data monitoring: formal committee	#21a	Composition of data monitoring committee (DMC); summary of its role and reporting structure; statement of whether it is independent from the sponsor and competing interests; and reference to where further details about its charter can be found, if not in the protocol. Alternatively, an explanation of why a DMC is not needed	n/a
Data monitoring: interim analysis	#21b	Description of any interim analyses and stopping guidelines, including who will have access to these interim results and make the final decision to terminate the trial	n/a
Harms	#22	Plans for collecting, assessing, reporting, and managing solicited and spontaneously reported adverse events and other unintended effects of trial interventions or trial conduct	n/a
Auditing	#23	Frequency and procedures for auditing trial conduct, if any, and whether the process will be independent from investigators and the sponsor	n/a
**Ethics and dissemination**			
Research ethics approval	#24	Plans for seeking research ethics committee / institutional review board (REC / IRB) approval	15
Protocol amendments	#25	Plans for communicating important protocol modifications (eg, changes to eligibility criteria, outcomes, analyses) to relevant parties (eg, investigators, REC / IRBs, trial participants, trial registries, journals, regulators)	n/a
Consent or assent	#26a	Who will obtain informed consent or assent from potential trial participants or authorised surrogates, and how (see Item 32)	7, 15
Consent or assent: ancillary studies	#26b	Additional consent provisions for collection and use of participant data and biological specimens in ancillary studies, if applicable	n/a
Confidentiality	#27	How personal information about potential and enrolled participants will be collected, shared, and maintained in order to protect confidentiality before, during, and after the trial	15

For peer review only

Declaration of interests	#28	Financial and other competing interests for principal investigators for the overall trial and each study site	22
Data access	#29	Statement of who will have access to the final trial dataset, and disclosure of contractual agreements that limit such access for investigators	n/a
Ancillary and post trial care	#30	Provisions, if any, for ancillary and post-trial care, and for compensation to those who suffer harm from trial participation	n/a
Dissemination policy: trial results	#31a	Plans for investigators and sponsor to communicate trial results to participants, healthcare professionals, the public, and other relevant groups (eg, via publication, reporting in results databases, or other data sharing arrangements), including any publication restrictions	15
Dissemination policy: authorship	#31b	Authorship eligibility guidelines and any intended use of professional writers	n/a
Dissemination policy: reproducible research	#31c	Plans, if any, for granting public access to the full protocol, participant-level dataset, and statistical code	n/a
**Appendices**			
Informed consent materials	#32	Model consent form and other related documentation given to participants and authorised surrogates	n/a
Biological specimens	#33	Plans for collection, laboratory evaluation, and storage of biological specimens for genetic or molecular analysis in the current trial and for future use in ancillary studies, if applicable	n/a

The SPIRIT checklist is distributed under the terms of the Creative Commons Attribution License CC-BY-ND 3.0. This checklist was completed on 05. February 2020 using https://www.goodreports.org/, a tool made by the EQUATOR Network in collaboration with Penelope.ai

For peer review only
